# Comprehensive safety and toxicity analysis of 2,2’-Bipyridine derivatives in combating MRSA biofilm formation and persistence

**DOI:** 10.3389/fcimb.2025.1493679

**Published:** 2025-01-24

**Authors:** Priyanka, Mohini Sharma, Bhavna Vaid, Ram Bharti, Sachin Raut, R. S. Jolly, Neeraj Khatri

**Affiliations:** ^1^ IMTECH Centre for Animal Resources & Experimentation (iCARE), Council of Scientific and Industrial Research (CSIR)-Institute of Microbial Technology, Chandigarh, India; ^2^ Academy of Scientific and Innovative Research, Ghaziabad, India; ^3^ PG Department of Chemistry, Sri Guru Tegh Bahadur (SGTB) Khalsa College, Sri Anandpur Sahib, Punjab, India; ^4^ Council of Scientific and Industrial Research (CSIR)-Institute of Microbial Technology, Chandigarh, India

**Keywords:** 2,2’-bipyridine derivatives, MRSA biofilms, cytotoxicity, antibacterial activity, acute oral toxicity, ADMET

## Abstract

**Introduction:**

Methicillin-resistant *Staphylococcus aureus* (MRSA) infections have become arduous to treat due to their capacity to form biofilms, develop persistence, and exhibit significant antimicrobial resistance. These factors contribute to the complexity of managing MRSA infections and highlight the urgent need for innovative treatment strategies.

**Objectives:**

This endeavor aims to evaluate the safety of 2,2’-Bipyridine (2,2’-Bipy) derivatives and their antimicrobial, anti-biofilm, and anti-persister activities in treating MRSA Infections.

**Methods:**

Six derivatives were screened for their ADMET properties and tested for minimum inhibitory concentrations against various bacterial strains using agar well diffusion and broth dilution. Safety studies were conducted through hemolysis tests, cell viability assays, and *in vivo* acute oral toxicity examinations. Bactericidal mechanisms and biofilm disruption effects were analyzed using crystal violet staining and confocal microscopy assays. The murine thigh infection model was also used to investigate the *in vivo* efficacy.

**Results:**

All derivatives exhibited favorable physicochemical profiles and ADMET properties and are predicted to be safe based on their drug-like properties. *in vitro* studies demonstrated that derivatives are non-toxic to 3T3 L1, and *in vivo* studies confirmed their safety in mice at a dose of 300 mg/kg and their non-hemolytic nature against rabbit red blood cells. All compounds showed potent antibacterial activity against the tested bacteria, including the resistant MRSA strain 831. They inhibited biofilm formation and eradicated biofilms in a dose-dependent manner against MTCC 737 and MRSA 831, and they effectively eliminated MRSA persister cells, outperforming the reference antibiotic vancomycin. These derivatives were found to depolarize the mitochondrial membrane and accumulate intracellular reactive oxygen species. These derivatives significantly reduced the bacterial load in the murine thigh infection model.

**Conclusion:**

The study concluded that 2,2’-Bipy derivatives possess significant antimicrobial activity, are non-toxic, and are effective in inhibiting biofilm formation and killing persister cells.

## Graphical Abstract

Schematic illustration of the experimental protocol depicting different activities of 2,2’-Bipy derivatives.

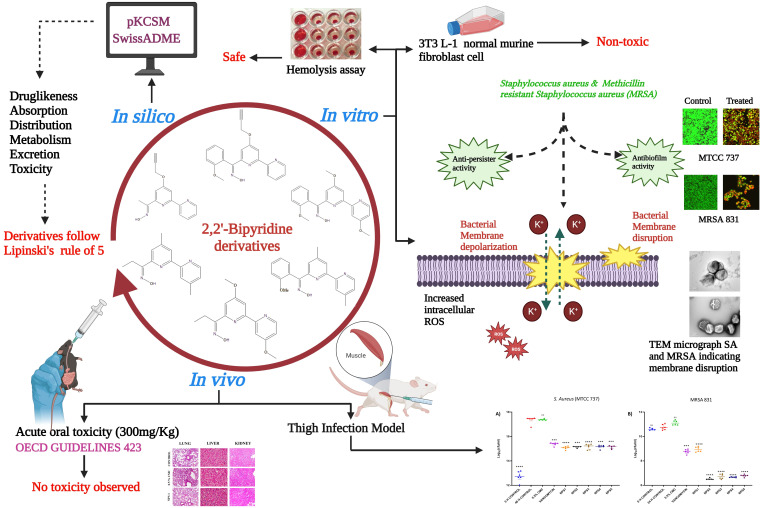

## 1 Introduction

According to WHO, Antimicrobial resistance (AMR) is among the top 10 worldwide public health hazards to humanity and develops when microbes evolve and stop responding to medications, making infections more difficult to cure and raising the risk of disease spread, serious illness, and death. The WHO has declared *Staphylococcus aureus (S. aureus)* as a “priority pathogen” due to its threat to public health (WHO, 2017). *S. aureus* is associated with a range of human illnesses, from superficial skin infections to potentially fatal systemic infections such as sepsis, endocarditis, pleuropneumonia, and osteoarthritis (Tong et al., 2015; Turner et al., 2019). Methicillin-resistant *S. aureus* (MRSA) is a subtype that is resistant to beta-lactam antibiotics such as penicillin, oxacillin, and amoxicillin (Vestergaard et al., 2019). The emergence of vancomycin-resistant strains led to failure rates of vancomycin treatment for MRSA infections (Shariati et al., 2020b). In both community and hospital settings, *S. aureus* is a significant opportunistic human pathogen that frequently causes disease (van Dalen et al., 2020). In addition to its ability to acquire resistance, they are able to evade antibiotics by switching to a non-growing dormant bacterial state, known as persisters, and evidence suggests that persisters are present in significant numbers in biofilms and are responsible for the relapse of biofilm-associated infections (Grassi et al., 2017; Kim et al., 2018a; Niu et al., 2024). Antibiotics targeting bacterial membranes bypass the reliance on cell permeability, making them effective against persisters, and can act as synergistic agents to enhance the efficacy of other antibiotics (Mehta et al., 2021; Zhang and Ma, 2019). The growing prevalence of multidrug-resistant strains of methicillin-resistant *S. aureus* underscores an urgent and unmet clinical need for innovative approaches to treat these infections (Miller et al., 2020).

2,2’-Bipyridine (2,2’-Bipy) is among the most critical and favored bidentate ligands and consists of two linked pyridine units, which bind to metal ions at two different sites via neutral nitrogen atoms (Constable and Housecroft, 2019). The 2,2’-Bipy ligand is particularly suitable as a metal chelating ligand because of its high redox stability and ease of functionalization (Kaes et al., 2000). Additionally, 2,2’-Bipy forms the distinct molecular scaffold of bioactive natural products such as caerulomycins (CAEs) and collismycins (COLs) (Pang et al., 2021). In previous studies by our collaborators, Caerulomycin A (Cae A) was isolated, purified, and characterized from *Actinoalloteichus spitiensis*, a novel species of actinomycete. A patent was filed for using 2,2’-Bipy compounds, and studies by our co-authors have demonstrated the immunosuppressive and iron-chelating properties of these derivatives in autoimmune and iron overload diseases (Singla et al., 2007, 2014; Gurram et al., 2014; Kaur et al., 2015; Jolly et al., 2018). Different 2,2’-Bipy complexes exhibit antiviral, antifungal, antimicrobial, and potent antitumor activities (Lee et al., 2017; Lahoum et al., 2019; Burgart et al., 2022; Nasiri et al., 2023). The biological evaluation of the bipyridine complexes exhibited antibacterial activity against MRSA strains, though only at high concentrations. However, the studies were limited, with a minimum investigation into biofilm inhibition, persistence, and resistance development and no assessment of *in vivo* efficacy in murine models (Wang et al., 2021; Aragoni et al., 2023). Therefore, we developed novel 2,2’-Bipy derivatives with ketoxime substituents instead of aldoximes, a class of compounds with >C-N=O-H moiety. Oxime moieties have been reported in the literature to have excellent antimicrobial properties with less toxicity (Yong Hong et al., 1997; Syed, 2014; Hughes, 2017; Kojo et al., 1988; Souza et al., 2016; Chauhan, 2013; Liu et al., 2014). The synthesis and characterization of these novel derivatives are comprehensively detailed in the PhD thesis of Dr. Bhavna Vaid, who is also a co-author of this manuscript (Vaid Bhavna, 2018). The key objective of this study is to evaluate the biological activity of these novel 2,2’-Bipy derivatives, hypothesizing that they are potential candidates for new drug therapies against various types of microbial infections, including drug-resistant strains.

## 2 Materials and methods

### 2.1 Chemicals

Potato Dextrose Agar (PDA), Nutrient Agar (NA), Mueller Hinton Broth (MHB), and Mueller Hinton Agar (MHA) were sourced from HiMedia. Dimethyl sulphoxide (DMSO), MTT [3-(4,5-dimethyl-thiazol-2-yl)-2,5-diphenyltetrazolium bromide], Polymyxin B, Valinomycin, DCFDHA (2′,7′-dichlorofluorescein diacetate, Sigma), Propidium iodide (PI), N-phenyl naphthylamine (NPN), Triton X-100 and Carboxy-methylcellulose (CMC) were procured from Sigma. Cell culture reagents 1X DMEM, 1X DPBS, Penicillin-Streptomycin (100 units), 0.25% Trypsin EDTA, and Foetal Bovine Serum (FBS) were acquired from GIBCO.

### 2.2 Cell culture

The murine fibroblast 3T3 L-1 cell line was procured from the National Centre for Cell Science (NCCS), Pune, India. The 3T3 L-1 cells were incubated at 37°C in an incubator maintained at 95% humidity and 5% CO_2_ in DMEM medium complemented with 10% fetal bovine serum (FBS) and 1% penicillin-streptomycin.

### 2.3 Bacterial strains and growth conditions

*Staphylococcus aureus* (MTCC 737), *Escherichia coli* (MTCC 739), *Pseudomonas aeruginosa* (MTCC 1934), *Micrococcus luteus* (MTCC 106), *Vibrio cholerae* (MTCC 3906) and *Klebsiella pneumoniae* (MTCC 618) were attained from Microbial Type Culture Collection (MTCC) of CSIR-IMTECH, Chandigarh, India. The MRSA 831, a clinical strain, was a kind gift from Dr. Hemraj Nandanwar, CSIR-IMTECH, Chandigarh. All bacterial strains were cultured overnight at 37°C on nutrient agar and experimental procedures were performed using MHB and MHA.

### 2.4 ADMET analysis

ADMET profiling (absorption, distribution, metabolism, excretion, and toxicity) of newly synthesized compounds and their derivatives must be determined before their use in preclinical and clinical trials (Agrwal et al., 2022; Alesawy et al., 2022). Numerous *in silico* tools and servers can predict the ADMET profiles of these chemical entities. Therefore, 2,2’-Bipy derivatives (NPS1-6) were screened based on crucial features, including the rules of Lipinski, Ghose, Veber, and Egan and their pharmacokinetic and toxic profiles using the web servers pkCSM (Pires et al., 2015) and SwissADME (Daina et al., 2017; Kharb et al., 2023; Kumar et al., 2023; Knoll et al., 2022).

### 2.5 Hemolytic activity

Blood from New Zealand White rabbits was collected in EDTA-coated vials. The blood was centrifuged at 1000 rpm and washed thrice with ice-cooled phosphate buffer saline (1X PBS, pH 7.4), and then resuspended at a 2% (v/v) concentration in PBS. The hemolytic assay was done using varying concentrations (0.156-400 µg/ml in two-fold dilutions) of NPS (1-6) bipyridine derivatives as described previously (Dubey et al., 2021). A known hemolytic agent, Triton-X 100, which lyses the RBCs, was taken as standard control, while 0.1% DMSO and PBS were used as vehicle and negative controls, respectively. 1X PBS without RBCs was used as the blank for this experiment, and absorbance was recorded at 570 nm. Percent hemolytic activity was calculated using a formula:


$$\text{Hemolysis (\%)=}\frac{\text{Absorbance (Test-Bank)}}{\text{Absorbance (Positive control-Bank)}}\times 100$$

### 2.6 Cytotoxicity (MTT assay)

Synthesized derivatives were evaluated for cytotoxicity in a 3T3-L1 cell line using the MTT assay (Ernawati et al., 2022). 8000 cells/well were added in 96-well tissue culture flat-bottom microtiter plates for 24 h and then incubated with synthesized molecules (0-400 µg/ml) for 48 h at 37°C in a CO_2_ incubator. DMSO was used as solvent at a concentration of 0.1% to ensure that any observed effects were not attributable to the solvent itself, whereas 1 µg/ml doxorubicin was used in toxicity tests as a positive control. Subsequently, the media was replaced with MTT solution (100 µl) diluted in fresh media (0.5 mg/ml), and incubated for 4 h. After incubation, 100 µl of stop solution (50% DMF with 20% SDS) was added to dissolve the formazan crystals, and the plates were further incubated for 1 h. Absorbance was measured at 570 nm using BioTEK Synergy multi-plate reader (Mosmann, 1983; Pratelli et al., 2023). Percent cell viability was calculated using the following formula:


$$\begin{array}{c} \text{Cell viability }\left(\%\right)=\\ \frac{\text{Absorbance }\left(\text{Untreated - blank}\right)\text{ - Absorbance }\left(\text{Test sample- blank}\right)}{\text{Absorbance }\left(\text{Untreated - blank}\right)}\times 100 \end{array}$$

### 2.7 Antibacterial activity

The antimicrobial activity of 2,2’-bipyridine derivatives was evaluated against a range of bacterial strains, including Gram-positive bacteria, *S. aureus* (MTCC 737), *M. luteus* (MTCC 106), and methicillin-resistant *S. aureus* (MRSA 831), as well as Gram-negative bacteria, *E. coli* (MTCC 739), *P. aeruginosa* (MTCC 1934), *V. cholerae* (MTCC 3906), and *K. pneumoniae* (MTCC 618). The assessments were conducted using the agar well diffusion method at a concentration of 50 µg/ml (Hassan et al., 2018). Clinical & Laboratory Standards Institute (CLSI) procedures were followed to determine minimum inhibitory concentrations (MICs) of the compounds in 96-well microplates against *S. aureus* (MTCC 737) and MRSA 831 using the broth-micro dilution assay in MHB (Ding et al., 2022; Sari et al., 2020). Briefly, 10 mg/ml master stocks and drugs were 2-fold diluted to final concentrations ranging from 0-200 µg/ml in the 96-well microtiter plate. Each well was then inoculated with an equal volume of bacterial suspension, having 1 × 10^5^ CFU/ml (0.01 OD_600_), and incubated for 18 h. Controls included media control, DMSO (0.5%), positive (vancomycin and ampicillin), and culture controls. The MICs of the derivatives were determined as the lowest concentrations at which no visual turbidity was observed.

### 2.8 Killing kinetics assay

MTCC 737 and MRSA 831 were grown in MHB medium overnight at 37°C with shaking at 200 rpm. In 96-well plates, cultures with a final concentration of 1 × 10^5^ to 1 × 10^6^ CFU/ml were added and incubated with 0.5 to 4 × MIC concentrations of 2,2’-Bipy derivatives. Untreated cells served as the negative control, while 0.5% DMSO and vancomycin were taken as vehicle and positive controls, respectively. The bacterial densities were recorded at OD_600_ at intervals of 2 h, starting from 0 h up to 18 h (Thakare et al., 2017; Thulshan Jayathilaka et al., 2021), and plotted as Log CFU/ml.

### 2.9 Biofilm inhibition and eradication assay

#### 2.9.1 Biofilm inhibition assay

MTCC 737 and MRSA 831 grown overnight and diluted to 0.01 OD with fresh MHB medium, with or without 2,2’-Bipy derivatives and vancomycin, to evaluate biofilm inhibition. In 96-well plates, 180 µl of culture (1 × 10^5^ to 1 × 10^6^ CFU/ml) was incubated at 37°C for 24 h with 0.5 - 4 × MIC of the derivatives. After incubation, cells were washed with 1X PBS, and stained with 100 µl of Crystal Violet dye for 15 min, and rinsed again with 1X PBS. The retained dye was dissolved in 100 µl of 95% ethanol, and absorbance was recorded at 595 nm (She et al., 2019).

#### 2.9.2 Biofilm inhibition imaging using confocal scanning laser microscopy

The bacterial cultures were grown using the procedure mentioned above (She et al., 2019). Biofilm adhesion was ensured by use of petri-dishes coated with poly-L-lysine. One milliliter of the bacterial suspension was dispensed into each polystyrene tissue culture plate (Himedia) on the coated side. The cultures were treated with 2,2’-Bipy derivatives (100 µg/ml) and vancomycin and incubated in a static environment for 24 h. Untreated wells were used as a control. After treatment, the media was disposed of, and the wells were washed three times with 0.85% saline. The wells were then stained for 35 to 40 min. Using SYTO 9 and PI dyes. Excess stain was removed by rinsing the wells three times, and the plates were dried in laminar airflow for a short while. Inverse confocal laser scanning microscopy (CLSM) [Nikon AI(R)] was used to examine the slides at 488 nm and 561 nm excitation and capture the representative images. NIS-Elements Viewer 5.21 was used to capture and process the images. Two independent experiments were done in triplicate.

#### 2.9.3 Biofilm eradication assay

For biofilm-eradication, 180 µl cultures were incubated in the 96-well plates at 37°C for 24 h to follow biofilm formation, and then 0.5 - 4 × MIC of the 2,2’-Bipy derivatives were added biofilm and incubated for another 24 h. Biofilm was washed with 1X PBS and analyzed using Crystal Violet staining following previously established protocol (She et al., 2019; Song et al., 2020).

### 2.10 Anti-persister activity

#### 2.10.1 Persister killing assay

To evaluate the persister-killing activity of 2,2’-Bipy derivatives, MTCC 737 and MRSA 831 cells were grown overnight to the stationary phase at 37°C (Kim et al., 2018b). The cultures were washed twice with 1X PBS, added to the 96-well plate with OD_600_ = 0.2, and incubated with 0.5 - 4 × MIC of 2,2’-Bipy derivatives with vancomycin used as positive control. The bacterial densities of persisters were recorded at OD_600_ at hourly intervals from 0 to 6 h.

#### 2.10.2 Biofilm persisters killing assay

To evaluate the effect of these derivatives on persister cells associated with the biofilm, a biofilm persister killing assay was executed, as reported by De Breij et al., 2018 with slight changes. MTCC 737 and MRSA 831 cells were grown to an OD_600_ of 0.5 at 37°C, then centrifuged at 2000 rpm, and diluted in fresh MHB medium containing 1 × 10^8^ CFU/ml. The same general procedure was followed as in the killing kinetics assay, and absorbance was recorded at 600 nm at hourly intervals up to 18 h. The number of live bacteria was determined depending on the absorbance readings.

### 2.11 Drug resistance screening

Drug resistance in MTCC 737 and MRSA 831 to 2,2’-Bipy derivatives was evaluated using sequential passage method. Cultures were grown overnight, adjusted to OD_600_ of 0.3 with MHB medium, and treated with MIC and 0.5 × MIC of all 2,2’-Bipy derivatives. After 24 h, absorbance was recorded. A 50 µl bacterial suspension was transferred to new 96-well plates with fresh medium and the same concentrations of derivatives. After 24 h again, absorbance was recorded, and the process was repeated every 24 h for 19 days (Sarkar et al., 2023). MICs were compared to the initial MIC, and a graph was plotted to illustrate the results.

### 2.12 Antibacterial mechanism

In our experiments, we found that NPS (1-5) derivatives exhibited excellent antibacterial activity. To further elucidate the mechanism behind their antibacterial potential, we conducted additional experiments.

#### 2.12.1 Transmission electron microscopy

MTCC 737 and MRSA 831 were cultured to an OD_600_ of 0.5 at 37°C, with shaking at 200 rpm. The cultures were then treated with 100 µg/ml 2,2’-Bipy derivatives and vancomycin (as the positive control) for 12 h. Untreated bacteria served as the negative control, while 0.5% of the DMSO was taken as vehicle control. After treatment, the bacterial cultures were centrifuged, dissolved in fresh 1X PBS (pH 7.4), loaded onto a carbon grid, and further processed for transmission electron microscopy (She et al., 2020).

#### 2.12.2 PI uptake assay

MTCC 737 and MRSA 831 were grown, washed, and resuspended in 5 mM HEPES (pH 7.2), with the OD_600_ normalized to 0.5. The cultures were then treated with 100 µg/ml of 2,2’-Bipy derivatives for 1 h. Polymyxin B (10 μM), a membrane destabilizer, was used as a reference control. Drug-free and vehicle controls (cells treated with 0.5% DMSO) were included to analyze changes in fluorescence. After 1 h of treatment, cultures were centrifuged and stained with 5 µM PI dye for 15 min. Following staining, the cultures were washed twice to remove the extra dye, and fluorescence was recorded at 485 nm excitation and 525 nm emission wavelengths (Zhou et al., 2020).

#### 2.12.3 N-phenyl naphthylamine (NPN) uptake assay

An N-phenyl naphthylamine (NPN) absorption assay was performed to assess the permeability of the inner membrane. MTCC 737 and MRSA 831 cultures were grown and diluted to an OD_600_ of 0.05. The NPN dye (8 µM) was added to the cultures and incubated with 100 µg/ml of 2,2’-Bipy derivatives for 15 min. Controls included Triton X- 100 (0.1%) as positive control and DMSO (0.1%) as a negative control. Fluorescence data were recorded with an excitation of 340 nm and an emission wavelength of 405 nm (Elliott et al., 2020).

#### 2.12.4 Membrane depolarization assay

The membrane depolarization assay was conducted as described by (Zhou et al., 2020) with some modifications. MTCC 737 and MRSA 831 cells in the mid-log growth phase were washed and resuspended in 5 mM HEPES buffer, with the absorbance adjusted to 0.05. The cells were then incubated with 0.1 M KCl and 0.4 µM Disc3(5) (Invitrogen) at room temperature in the dark for 1 h. The plates were then incubated with 100 μg/ml 2,2’-Bipy derivatives for 30 min. Fluorescence intensity was measured with excitation and emission wavelengths of 622 nm and 670 nm, respectively. Valinomycin (16 µg/ml) and 0.1% DMSO (She et al., 2020) were used as positive and negative controls, respectively.

#### 2.12.5 Reactive oxygen species measurement

The experiment was conducted as reported previously (Song et al., 2020) with slight modifications. The level of reactive oxygen species (ROS) in MTCC 737 and MRSA 831, incubated with 2,2’-Bipy derivatives, was analyzed using DCFH-DA dye (2′,7′-dichlorofluorescein diacetate). MTCC 737 and MRSA 831 were grown overnight, washed, and resuspended in 1X PBS with OD_600_ of 0.05. The cultures were incubated with 0.4 μM DCFH-DA dye for 30 min. in the dark. After 30 min., 10 μl of 2,2’-Bipy derivatives (at a concentration of 100 µg/ml) were added to a black 96-well plate and incubated for an additional 30 min. Fluorescence intensity was measured at an excitation of 488 nm and an emission of 525 nm. Increased fluorescence corresponds to increased intracellular ROS levels compared to untreated cells and the vehicle control.

### 2.13 Animal studies

Female BALB/c and C57BL/6 mice, aged 6 to 8 weeks (19-25 g), were acclimatized for one week prior to experiments. Animals were housed in individually ventilated cages with controlled humidity (30-70%), a photoperiod of 12:12, and temperature was maintained at 24-25°C. Food pellets and sterile water were available at all times. All the procedures on animals were done following the guidelines of the Committee for the Control and Supervision of Experiments on Animals (CCSEA), India. The use of mice for acute oral toxicity studies and the murine thigh infection model, as well as rabbit blood for hemolysis assays, was approved by the Institutional Animal Ethics Committee (IAEC) of the CSIR-Institute of Microbial Technology, Chandigarh, under protocol numbers IAEC/19/19 and IAEC/22/07.

#### 2.13.1 Acute oral toxicity in mice

Acute oral toxicity was performed in mice to evaluate the *in vivo* toxicity of 2,2’-Bipy derivatives according to OECD guideline 423 (OECD, 2001). In this study, a starting dose of 300 mg/kg body weight was used, as the derivatives were insoluble at 2000 mg/kg. Mice (n=3) were divided into eight groups - Group 1 (control), Group 2 (vehicle), and Groups 3-8 (2,2’-Bipy derivatives NPS1, NPS2, NPS3, NPS4, NPS5, and NPS6, respectively). Prior to dosing, animals were fasted for 3-4 h. A single dose of 2,2’-Bipy derivatives (dissolved in 0.5% CMC) was administered orally by gavage and post-dosing, the mice were kept fasted for an additional 1- 2 h. According to OECD guideline 423, symptoms and behavior such as alterations in the mucous membranes, eyes, skin and fur, respiration, salivation, diarrhea, tremors, convulsions, sleep, coma, lethargy, and death were observed. After treatment, the toxic symptoms, time of onset, duration of recovery, and death of the animals were observed initially for 4 h and then daily for up to 14 days. Body weight was recorded weekly. On day 15, blood was collected by retroorbital method for hematological as well as biochemical analysis. and mice of different groups were euthanized by cervical dislocation after deep isoflurane anesthesia, and a necropsy was performed. Vital organs, including the brain, lungs, heart, liver, spleen, and kidneys, were collected for histological analysis. These organs were fixed in 10% neutral buffered formalin, sectioned, and further embedded in paraffin wax before the tissues were cut into 5 micron sections and stained with hematoxylin and eosin (H&E). Histopathological examination was performed under a light microscope.

#### 2.13.2 Thigh infection model in mice

The efficacy of 2,2’-Bipy derivatives against MTCC 737 and MRSA 831, a clinical isolate, was investigated using a thigh infection model in mice. Female BALB/c mice (n=6) were administered with two doses of cyclophosphamide, 150 mg/kg and 100 mg/kg, administered 4 days and 1 day prior to infection to induce neutropenia. The mice were then infected intramuscularly in the right thigh muscle with 1 × 10^6^ CFU/ml MTCC 737 and 1 × 10^8^ CFU/ml MRSA 831. In MTCC 737 infected group, all mice received an oral dose of 2,2’-Bipy derivatives (30 mg/kg) and vancomycin (30 mg/kg) 4 h after infection. Vehicle and control groups were treated with 0.5% CMC and saline, respectively. After 4 h of infection, one of the control group of mice was sacrificed, and thigh muscle was collected. The muscle tissue was homogenized, and the CFU in the muscle was determined by spreading the homogenate on MHA plates using serial dilution in PBS. After 48 h, the remaining mice from all groups were sacrificed, and CFU per thigh was calculated (Ling et al., 2015). A similar experiment was performed with the MRSA 831 group, where treatment was given 2 h after infection, and the mice were sacrificed after 24 h of treatment (Choi et al., 2020).

### 2.14 Statistical analysis

Data analysis was done using GraphPad Prism software (version 9.2), p-values were analyzed using Student’s-tests for comparisons between two-groups and one-way or two-way ANOVA tests for comparisons among multiple groups. Each experiment included three biological replicates, with three technical replicates. Data are presented as Mean ± SD. Statistical significance was defined as *p<0.05, and **p<0.01, ***p<0.001 and ****p<0.0001.

## 3 Results

### 3.1 Chemical structures

2,2’-Bipy derivatives were synthesized using commercially available 2,2’-bipyridine (Sigma). Oxime moieties enhancing the functionality of antibiotics exhibit cytotoxic properties, making them effective against bacterial, fungal, and viral infections. One such example is Ceftobiprole medocaril, where oximes can address resistance issues and enhance efficacy. This underscores the importance of oxime modifications in advancing antibiotic therapies and overcoming resistance challenges (Hughes, 2017) and, alkyl or aryl groups were added to the oxime moiety further to modulate hydrophobicity of the molecule. Allyloxy and propargyloxy groups enhance antibacterial activity by improving compounds lipophilicity and targeting bacterial respiration, demonstrating effectiveness against resistant strains like MRSA and VRE, which exhibit low cytotoxicity, making them promising candidates for next-generation antimicrobials (Li et al., 2016; Kisiel-Nawrot et al., 2022). The molecules used in this study are NPS1 (N-[1-(4-allyloxy[2,2’-bipyridin]-6-yl)ethyldiene]hydroxylamine), NPS2 (N-[(4-propargyloxy-[2,2’-bipyridin]-6-yl)(2-methoxyphenyl) methylidene] hydroxylamine), NPS3 (N-[(4,4’-dimethoxyl[2,2’-bipyridin]-6-yl)(2-methoxyphenyl) methylidene] hydroxylamine), NPS4 (N-[(4,4’-dimethyl[2,2’-bipyridin]-6-yl)(2-methoxyphenyl) methylidene] hydroxylamine), NPS5 (N-[1-(4,4’-dimethoxy[2,2’-bipyridin]-6-yl)propyldiene]hydroxylamine), and NPS6 (N-[1-(4,4’-dimethoxy[2,2’-bipyridin]-6-yl)propyldiene]hydroxylamine) respectively with the structures shown in **Supplementary Figure S1**.

### 3.2 *In silico* studies

*In silico* analysis was performed to corroborate the physiochemical and ADMET (absorption, distribution, metabolism, excretion, and toxicity) properties of 2,2’-Bipy derivatives.

#### 3.2.1 Drug likeness

Drug likeness was assessed based on Lipinski’s Rule of 5, which considers molecular weight, hydrogen bond acceptors, hydrogen bond donors, and log P (octanol/water partition coefficient). According to this rule, compounds should have a molecular weight less than 500 Da, hydrogen bond acceptors ≤10, hydrogen bond donors ≤5, and log P ≤5. All 2,2’-Bipy derivatives complied with Lipinski’s rules, as well as those of Ghose, Egan, and Veber, except vancomycin, which exceeds the molecular weight threshold and has higher hydrogen bond donors and acceptors. Ampicillin did not meet Egan’s rule. A summary of these findings is presented in **Table 1**. Although predictions are not always experimentally validated, the calculated masses of these derivatives closely matched the experimental values obtained via mass spectrometry.

**Table 1:** Physicochemical and drug-likeness properties of 2,2’- Bipy derivatives based on SwissADME and pKCSM.

Compounds	Physiochemical properties						Violations of				Synthetic accessibility
							Lipinski’s	Veber’s	Egan’s	Ghose’s	
	Molecular weight (g/mol)	Molar refractive index	Rotatable bonds	Log P (Octanol/ Water)	H AD	H-BD					
**Rules**	**≤ 500**	**40 ≤ MR ≤ 130**	**< 10**	**< 5**	**≤ 10**	**< 5**	**≤ 1**	**Y/N**	**Y/N**	**Y/N**	**0 < SA < 10**
**NPS1**	267.28	75.92	4	2.17	5	1	0	Y	Y	Y	2.96
**NPS2**	359.38	102.1	6	3.18	6	1	0	Y	Y	Y	3.4
**NPS3**	365.38	100.81	6	2.85	7	1	0	Y	Y	Y	3.37
**NPS4**	333.38	97.76	4	3.52	5	1	0	Y	Y	Y	3.21
**NPS5**	287.31	79.45	5	2.14	6	1	0	Y	Y	Y	3.07
**NPS6**	255.31	76.39	3	2.87	4	1	0	Y	Y	Y	2.94
**Vancomycin**	1449.71	–	13	0.10	25	19	4	N	N	N	4.16
**Ampicillin**	349.4	92.56	5	0.08	5	3	0	Y	N	Y	4.16

Bold values depict standard rules for physiochemical properties.

#### 3.2.2 ADMET prediction

ADMET profiles for the 2,2’-Bipy derivatives were analyzed using the pkCSM tool (**Table 2**). The derivatives showed over 90% intestinal absorption, suggesting effective oral bioavailability. They also met the threshold for effective transdermal delivery with log Kp values <-2.5. Water solubility ranged from 0 to -4.203, which is favorable for good absorption and distribution.

**Table 2:** ADMET properties of 2,2’-Bipy derivatives.

Property	Parameter/Model name (Unit)	NPS1	NPS2	NPS3	NPS4	NPS5	NPS6	Ampicillin	Vancomycin
Absorption	Water solubility (log mol/L)	-3.58	-3.521	-3.393	-4.203	-3.733	-3.169	-2.396	-2.892
	Skin Permeability (log Kp)	-2.76	-2.75	-2.744	-2.744	-2.735	-2.62	-2.735	-2.735
	Intestinal absorption (% Absorbed)	97.55	98.214	96.653	96.044	96.336	95.551	43.034	0
	P-glycoprotein I inhibitor	No	Yes	Yes	Yes	No	No	No	No
	P-glycoprotein II inhibitor	No	No	No	No	No	No	No	No
Distribution	BBB permeability (log B.B.)	-0.407	-0.659	-0.904	-0.432	-0.676	0.432	-0.767	-3.351
	VDss (Human) (log L/kg)	-0.065	0.294	-0.043	0.067	-0.051	0.199	-1.23	-0.284
Metabolism	CYP1A2 inhibitor	Yes	Yes	Yes	Yes	Yes	Yes	No	No
	CYP2C9 inhibitor	No	No	No	No	No	No	No	No
	CYP3A4 inhibitor	No	Yes	No	Yes	No	No	No	No
	CYP2D6 inhibitor	No	No	No	No	No	No	No	No
	CYP2C19 inhibitor	No	Yes	Yes	Yes	No	Yes	No	No
Excretion	Renal OCT2 substrate	No	No	No	Yes	No	No	No	No
	Total Clearance (log ml/min/kg)	0.811	0.804	0.747	0.592	0.826	0.735	0.337	-1.273
Toxicity	Max tolerated dose (Human) (log mg/kg/day)	0.429	-0.065	0.144	-0.114	0.556	0.488	0.952	0.439
	Oral Rat Acute Toxicity(LD50)(mol/kg)	2.711	3.242	3.244	2.984	2.689	2.829	1.637	2.482
	Oral Rat Chronic Toxicity (log mg/kg_bw/day)	1.55	1.277	1.139	0.956	1.003	1.545	2.398	9.212
	hERG I inhibitor	No	No	No	No	No	No	No	No
	hERG II inhibitor	No	Yes	No	Yes	No	No	No	Yes
	Skin Sensitization	No	No	No	No	No	No	No	No
	Hepatotoxicity	Yes	Yes	Yes	Yes	Yes	Yes	Yes	Yes
	AMES toxicity	No	No	No	No	No	No	No	No
	Pyriformis toxicity (log µg/L)	0.281	0.299	0.289	0.33	0.292	0.352	0.285	0.285
	Minnow toxicity (log mM)	2.391	2.054	1.601	1.674	2.177	1.059	4.232	14.167

P-glycoprotein interaction varied among the derivatives: NPS1, NPS5, and NPS6 had no effect, while NPS2, NPS3, and NPS4 inhibited P-glycoprotein I but not P-glycoprotein II. This selective inhibition influenced the overall effect on P-glycoprotein, as determined by the *in silico* absorption analysis. The volume of distribution (VDss) for all derivatives, except vancomycin and ampicillin, fell between -0.15 and 0.45 log L/kg, indicating balanced tissue distribution. All derivatives, including ampicillin, crossed the blood-brain barrier (log BB >-1.0), unlike vancomycin (log BB -3.351).

Regarding cytochrome P450 interactions, the ADMET analysis revealed that all 2,2’-Bipy derivatives inhibit CYP 1A2, which plays a significant role in the metabolism of various drugs. This inhibition could potentially affect the pharmacokinetics of co-administered drugs that are substrates of CYP 1A2. Importantly, the derivatives were non-inhibitors of CYP 2D6 and CYP 2C9, which are involved in the metabolism of a wide range of drugs, suggesting that these compounds have a lower likelihood of causing drug-drug interactions mediated by these enzymes. Furthermore, NPS2 and NPS4 were found to inhibit both CYP 2C19 and CYP 3A4, two isoforms involved in metabolizing numerous therapeutic agents. While this interaction could suggest potential metabolic interference with drugs processed by these enzymes, the selective inhibition of these isoforms may be leveraged to enhance the efficacy of the derivatives in specific therapeutic contexts. The fact that these interactions are selective further supports the potential of the 2,2’-Bipy derivatives as candidates for drug development with tailored metabolic profiles.

The derivatives showed higher overall clearance rates than vancomycin and ampicillin and did not inhibit renal OCT2. AMES toxicity tests indicated no mutagenic potential, and none of the derivatives inhibited hERG channels, making them suitable for topical application.

### 3.3 *In vitro* studies

#### 3.3.1 Hemolysis assay

**Figure 1:**
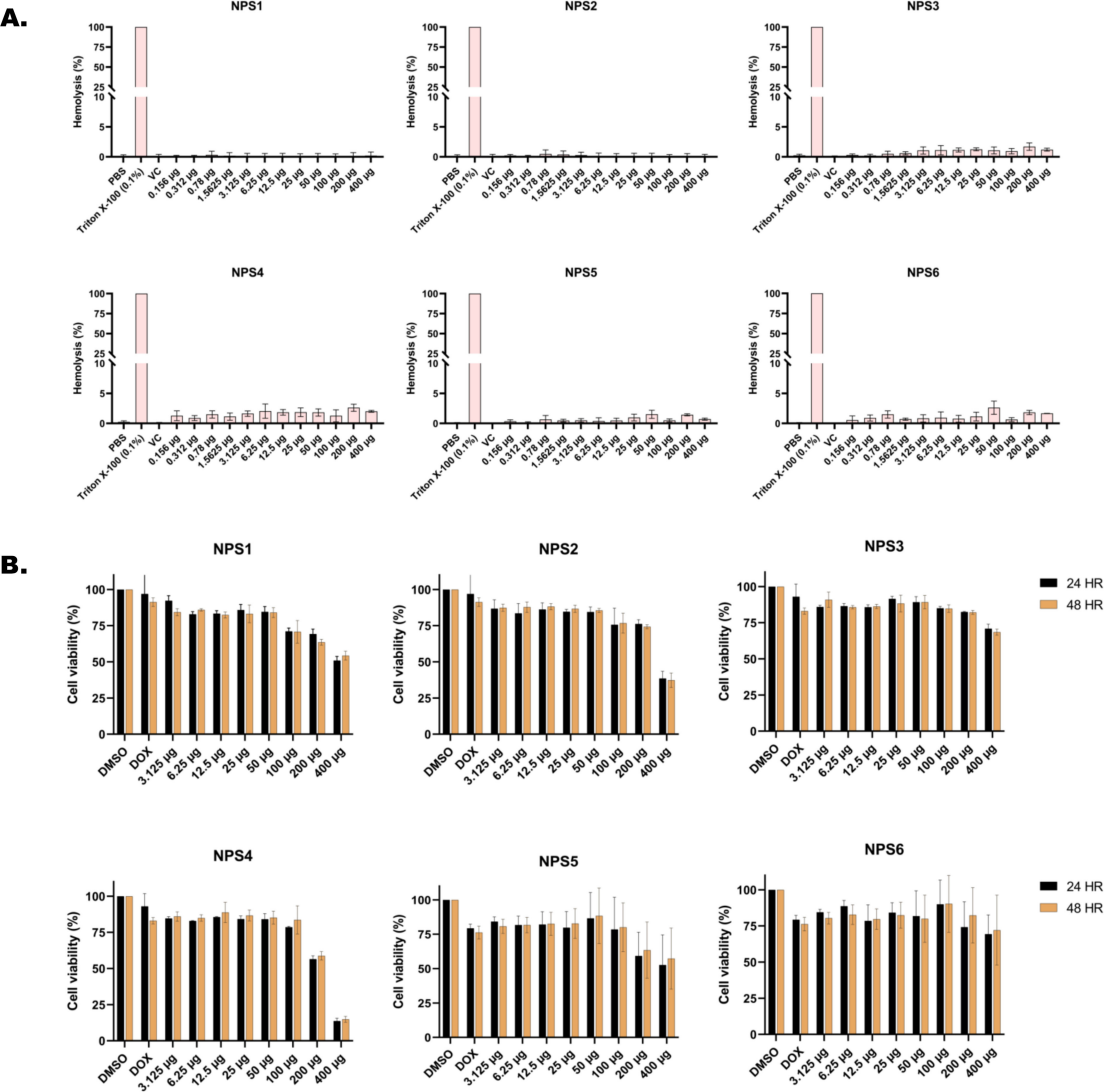
*In vitro* toxicity assessment of 2,2’-Bipy derivatives **(A)** Hemolytic assay against rabbit RBCs (Triton X-100 was used as positive control) **(B)** Cell viability assay on 3T3L-1 cell line (normal murine fibroblast cell line) at 24 h and 48 h using MTT assay (Doxorubicin and DMSO-treated cells were considered positive and negative controls). The cumulative data of three independent experiments (n=3) was plotted in the graph, and data is represented as Mean ± SD.

The ability of all six derivatives (NPS1-6) to lyse RBCs was assessed using hemolysis assay. In this assay, none of the derivatives exhibited hemolysis of rabbit red blood cells across a concentration range of up to 400 μg. In contrast, the positive control, Triton X-100, showed severe hemolysis at a concentration of 0.1% (**Figure 1A**). Therefore, findings of hemolytic assay revealed that all 2,2’-Bipy derivatives are non-hemolytic/non-toxic.

#### 3.3.2 Cytotoxicity assay in normal murine fibroblast cell line (3T3L-1 cell line)

Cytotoxicity of the 2,2’-Bipy derivatives was assessed using an MTT assay on 3T3L-1 cells. All derivatives exhibited more than 75% cell viability at 24 h and 48 h (**Figure 1B**). However, at a concentration of 400 μg/ml, the viability decreased to 50% or less. The calculated IC_50_ (µg/ml) values differed among derivatives and were 151.2, 1712, 253, 278.8, 151.5, and 168.1 for 24 h, and 137.6, 1341, 274.3, 239.6, 157.2, and 685.4 at 48 h for NPS1, NPS2, NPS3, NPS4, NPS5, and NPS6.

#### 3.3.3 Antimicrobial activity

**Figure 2:**
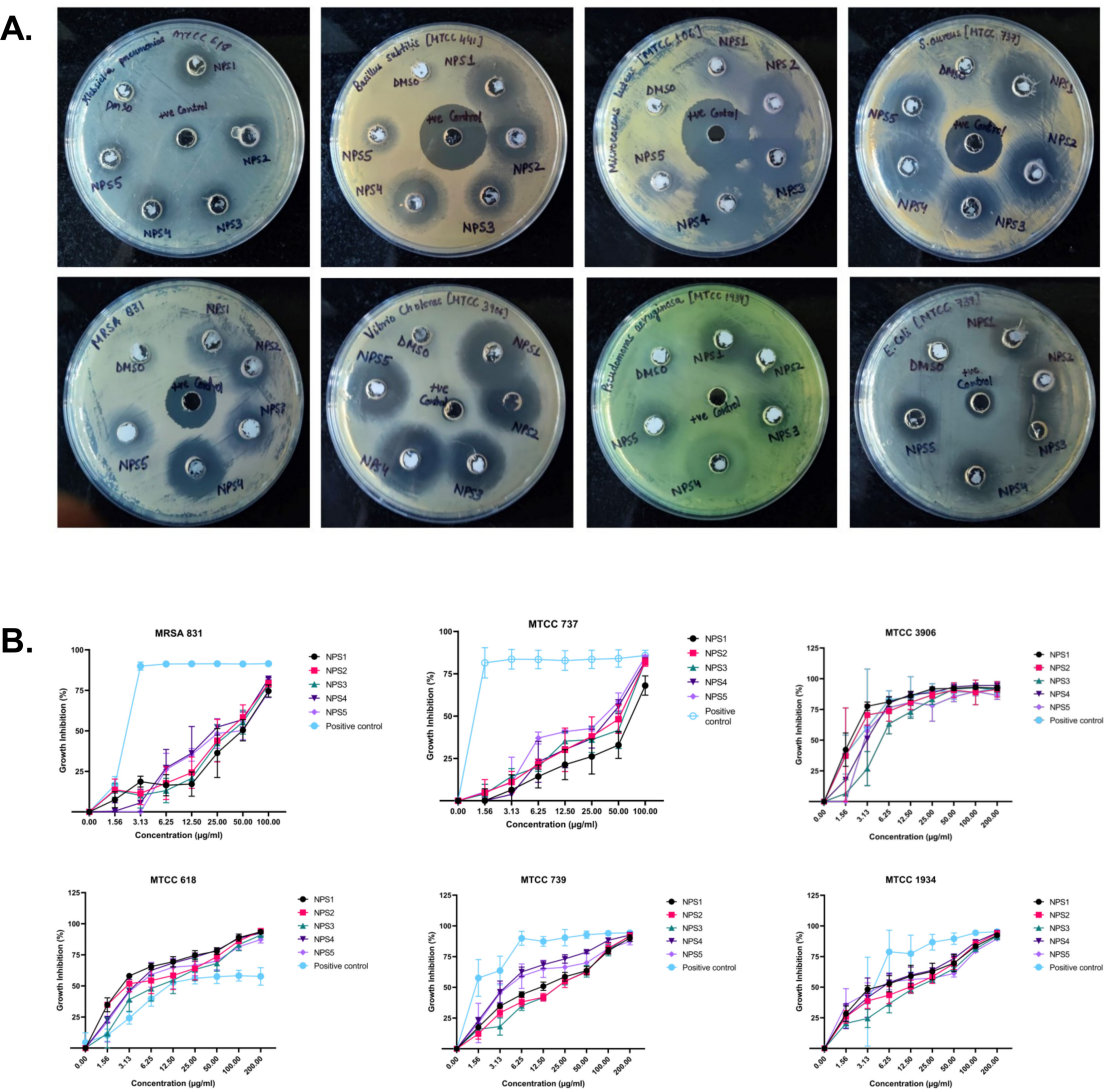
Antibacterial activity of 2,2’-Bipy derivatives **(A)** Representative images of Agar well diffusion assay against Gram-positive and Gram-negative bacteria, **(B)** Growth inhibition of bacteria. Vancomycin, Ampicillin, and PBS were included as the reference control. The cumulative data of three independent experiments (n=3) was plotted in the graph, and data is represented as Mean ± SD.

The antimicrobial activity of NPS1-NPS5 was evaluated against battery of strains including Gram-positive as well as Gram-negative strains as mentioned in section 2.7, using the agar well diffusion method (**Figure 2A**). The zone of inhibition for the inhibitory activity of NPS (1-5) at 50 µg/ml was measured in millimeters (mm) and is presented in **Table 3**. At this concentration, NPS6 exhibited poor diffusion due to its low solubility. NPS4 and NPS5 showed the highest zone of inhibition (14 mm) against MRSA 831. The minimum inhibitory concentrations (MICs) were determined using broth microdilution assay, and it was confirmed that all derivatives were effective against the tested bacteria (**Table 4**). NPS1 and NPS4 were found to be more effective than positive control against *V. cholerae*, and all of them showed activity against clinical MRSA 831. Therefore, we decided to proceed with *S. aureus* and MRSA 831 for further experiments. The therapeutic index (TI) calculated from 24-hour IC_50_ values was ≥3, indicating the safety of these derivatives (**Figure 2B**).

**Table 3:** Zone of inhibition (in mm) of 2,2’-bipyridine derivatives against various microbial strains.

Compounds	NPS1	NPS2	NPS3	NPS4	NPS5	Positive Control
*M. luteus* (MTCC 106)	ND	17.00 ± 0.00	14.67 ± 1.15	19.33 ± 0.58	7.33 ± 2.89	21.00 ± 0.00^*^
*B. subtilis* (MTCC 441)	5.33 ± 2.31	8.33 ± 1.15	4.67 ± 1.15	10.00 ± 1.00	4.00 ± 0.00	19.33 ± 0.58^*^
*K. pneumoniae* (MTCC 618)	6.00 ± 3.46	ND	ND	ND	13.00 ± 1.00	ND^*^
*S. aureus* (MTCC737)	17.00 ± 2.65	14.00 ± 0.00	13.67 ± 1.15	19.00 ± 0.00	10.00 ± 1.73	14.00 ± 0.00^#^
*E. coli* (MTCC739)	9.33 ± 0.58	9.00 ± 0.00	ND	9.00 ± 1.00	9.00 ± 1.00	4.00 ± 0.0^*^
MRSA 831 (MRSA)	8.67 ± 0.58	9.67 ± 0.58	9.67 ± 0.58	14.33 ± 0.58	14.33 ± 0.58	12.00 ± 0.00^#^
*P. aeruginosa (*MTCC1934)	7.67 ± 1.53	8.33 ± 1.53	11.33 ± 1.15	18.00 ± 1.00	18.00 ± 1.00	ND^*^
*V. cholerae* (MTCC 3906)	15.33 ± 1.53	13.00 ± 1.73	12.33 ± 2.52	17.67 ± 3.21	17.67 ± 3.21	ND^*^

ND, Not detected; Ampicillin^*^ and Vancomycin^#^ has been used as a positive control against Gram-negative and Gram-positive bacteria.

**Table 4:** Minimum inhibitory concentration values (µg/ml) of 2,2’-Bipy derivatives and standard controls against bacterial strains.

Compounds	NPS1	NPS2	NPS3	NPS4	NPS5	Positive Control
***E. coli* (MTCC739)**	>100	> 100	> 100	> 100	> 100	6.25^*^
***V. cholerae* (MTCC 3906)**	12.5	25	25	12.5	50	25^*^
***P. aeruginosa (*MTCC1934)**	>100	> 100	> 100	> 100	> 100	25^*^
***S. aureus* (MTCC737)**	50	50	50	50	50	6.25^#^
***K. pneumoniae* (MTCC 618)**	>100	> 100	> 100	> 100	> 100	> 100^*^
**MRSA 831**	50	50	50	50	50	6.25^#^

Vancomycin^#^ and Ampicillin^*^ has been used as a positive control against Gram-negative and Gram-positive bacteria.

#### 3.3.4 Time kill-kinetics

**Figure 3:**
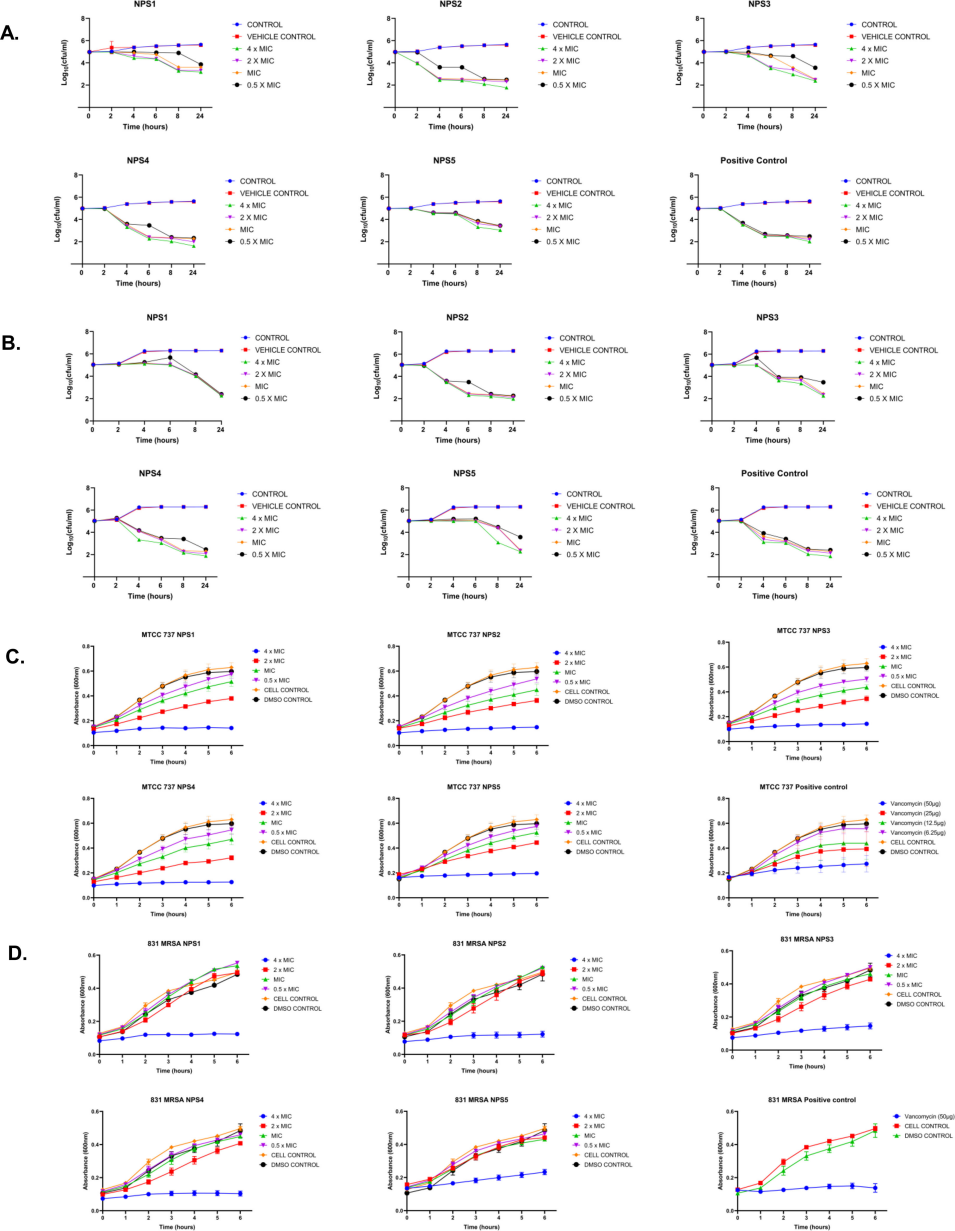
Time kill kinetics of **(A)** MTCC 737 and **(B)** MRSA 831 at four different concentrations 0.5 - 4 × MIC of 2,2’-Bipy derivatives and vancomycin (Positive control). Persister kill kinetics of **(C)** MTCC 737 and **(D)** MRSA 831 at four different concentrations 0.5- 4 X MIC of 2,2’-Bipy derivatives and vancomycin (Positive control). Control (untreated cells) and vehicle control were used to monitor normal growth. The cumulative data of three independent experiments (n=3) was plotted in the graph, and data is represented as Mean ± SD.

The derivatives exhibit antibacterial activity against *S. aureus* and MRSA 831. A time kill-kinetics assay was performed to evaluate their bactericidal effect. The assay demonstrated that derivatives NPS (1-5) were bactericidal against both *S. aureus* and MRSA 831, showing a dose- and time-dependent effect at concentrations of 0.5 to 4 × MIC. Significant reductions in bacterial counts, from 6 to 2.6-2 Log10 CFU/ml, were observed after 2 h of treatment (**Figures 3A, B**).

#### 3.3.5 2,2’-Bipy derivatives show activity against persister cells

A time-kill kinetics assay was conducted on *S. aureus* and MRSA 831 persister cells, both of which have high biofilm-forming potential. At a 4-fold MIC, the derivatives effectively reduced persister cell counts compared to vancomycin at the same concentration. Treatment with NPS3 and NPS4 with a 4-fold MIC significantly reduced the number of persister cells in the MRSA strain. Although the same concentration of derivatives decreased persister cell counts in *S. aureus*, in contrast, vancomycin showed similar activity only at a concentration of 100 µg/ml (**Figures 3C, D**).

#### 3.3.6 Biofilm inhibition and eradication

**Figure 4:**
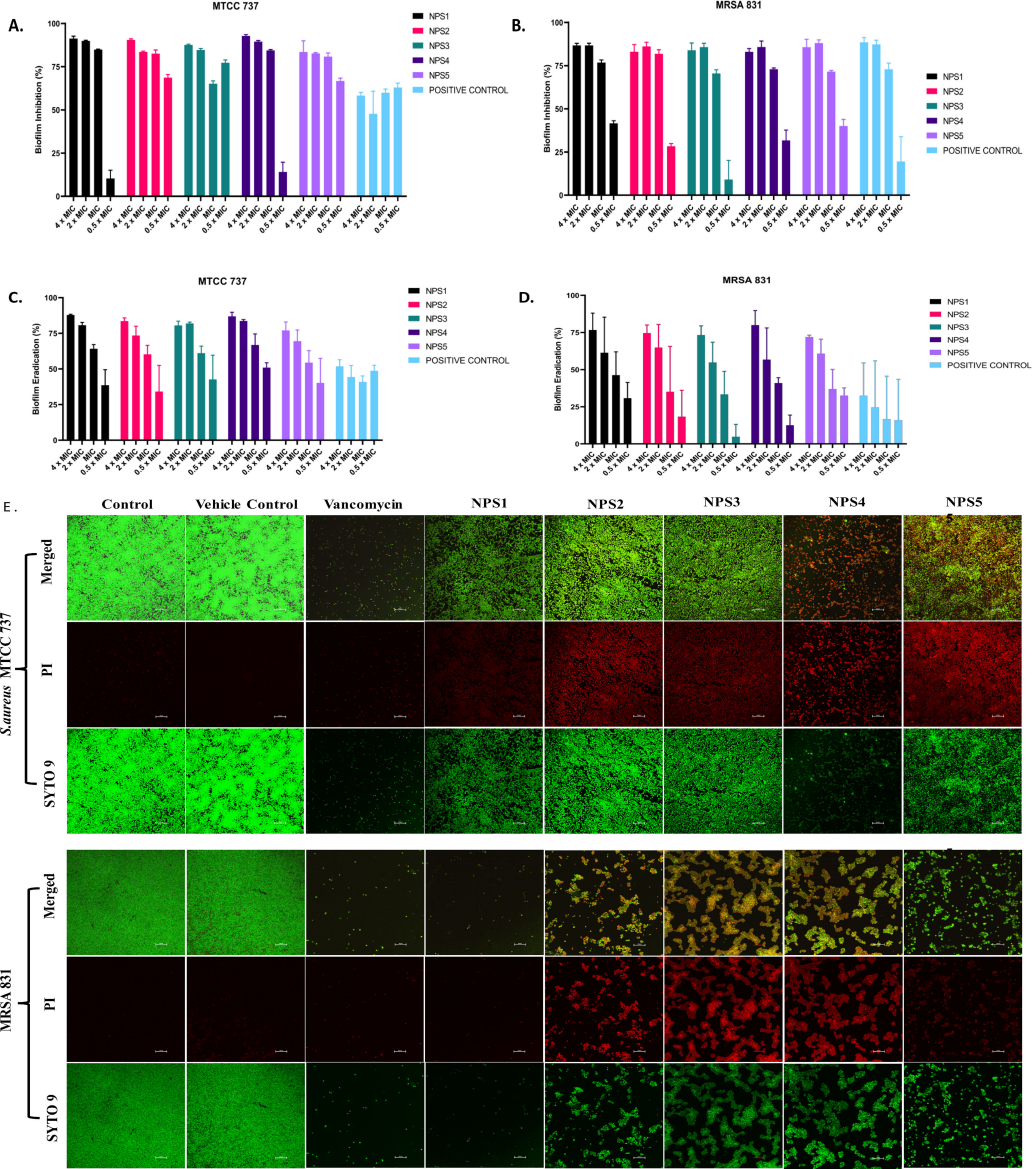
Effect of 2,2’-Bipy derivatives on biofilms. **(A, B)** Biofilm inhibition against MTCC 737 and MRSA 831; **(C, D)** Biofilm eradication against MTCC 737 and MRSA 831 at four different concentrations 0.5 - 4 × MIC of 2,2’-Bipy derivatives and vancomycin. The cumulative data of three independent experiments (n=3) was plotted in the graph, and data is represented as Mean ± SD. **(E)** Representative CLSM images of MTCC 737 and MRSA 831 after incubation with SYTO 9 (green fluorescence) and PI (red fluorescence) indicate the live and dead cells. Two independent experiments were performed in triplicate.

2,2’- Bipy have been shown to inhibit the growth of *S. aureus* and MRSA 831. Additionally, we aimed to test the inhibitory potential of these derivatives against biofilm formation. Biofilms, which are collections of microorganisms adhering to a surface matrix pose significant health risks by increasing antibiotic resistance and causing severe infections in humans. We evaluated the anti-biofilm activity of 2,2’-Bipy derivatives against Gram-positive bacteria (MTCC 737, MRSA 831) and Gram-negative bacteria (MTCC 739, MTCC 1934, MTCC 3906, and MTCC 618) at concentrations ranging from 0.5 to 4 times the MIC. These derivatives inhibited biofilm formation in all strains in a dose-dependent manner, achieving up to 75% inhibition at 4 × MIC (**Figures 4A, B**; **Supplementary Figures S2A–D**), indicating effectiveness of these derivatives. NPS1 and NPS4 are highly effective against *S. aureus* when used at MIC or higher concentrations, while the remaining derivatives exhibited 50% inhibition at half their respective MICs. When tested against resistant strain MRSA 831, all the derivatives showed inhibition comparable to vancomycin, highlighting their potent anti-staphylococcal activity. Further, to check if these derivatives are effective in disrupting a pre-formed biofilm, a biofilm eradication assay was conducted using MTCC 737, MRSA 831, MTCC 1934, and MTCC 3906. The derivatives NPS (1-5) effectively eradicated the biofilms in a dose-dependent manner (**Figures 4C, D**; **Supplementary Figures S3A, B**). As depicted in the figures, all derivatives show up to 75% eradication of pre-formed biofilms of *S. aureus* and demonstrated increased efficacy against MRSA biofilms compared to the positive control, vancomycin. These results highlight the potential of these derivatives as alternatives to traditional antibiotics for treating resistant bacterial strains. Furthermore, the findings confirmed the efficacy of 2,2’-Bipy derivatives against *Staphylococcus* biofilms, as well as their significant anti-biofilm activity against both Gram-positive and Gram-negative bacteria.

**Figure 5:**
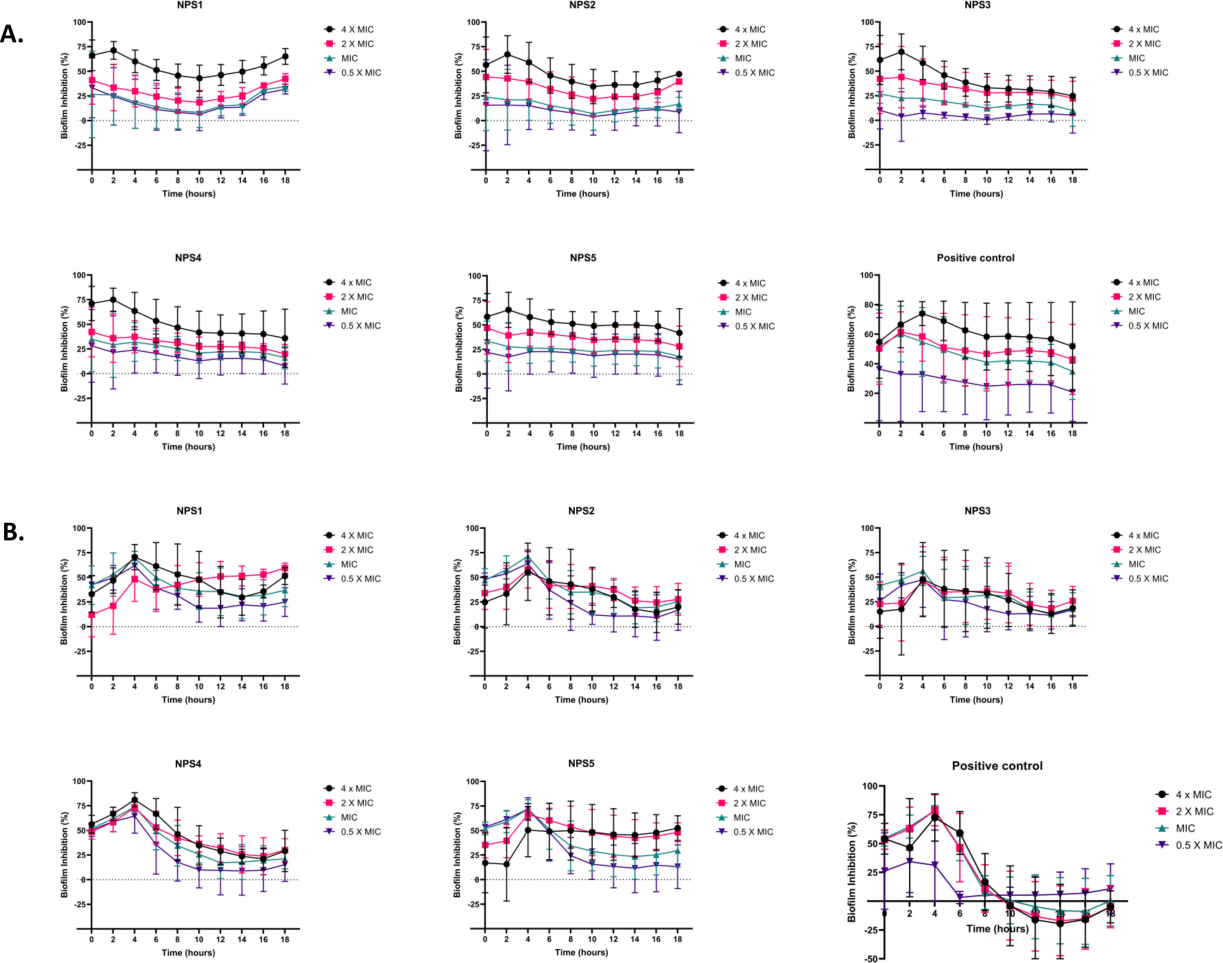
Effect of 2,2’-Bipy derivatives in inhibiting persister’s biofilm developed by **(A)** MTCC 737 and **(B)** MRSA 831, at four different concentrations 0.5 - 4 × MIC of 2,2’-Bipy derivatives and vancomycin. Data is represented as Mean ± SD and the cumulative data of three independent experiments (n=3) was plotted in the graph.

A CLSM assay was performed to visualize the effect of these derivatives on the biofilms of MTCC 737 and MRSA 831 using SYTO 9 dye and propidium iodide. SYTO 9 is a fluorescent dye that stains living and damaged cells, while propidium iodide penetrates the dead or membrane-compromised cells. In **Figure 4E**, the untreated and vehicle controls exhibited an intact biofilm lawn with high SYTO 9 and low PI fluorescence, indicating live cells and biomass of MTCC 737 and MRSA 831. Vancomycin, the positive control, showed a low number of cells, indicating effective biofilm disruption and severe cell damage. 2,2’-Bipy derivatives showed varying efficacy in inhibiting biofilms, of MTCC 737 and MRSA 831. In the biofilms of MTCC 737, NPS4 caused significant disruption and damage, while the other derivatives show only slight disruption. In MRSA 831, derivatives NPS (1-5) effectively disrupted the biofilms, with the remaining cells retaining both SYTO9 and PI dye, indicating membrane-damaged cells. The results demonstrated that NPS4 efficiently inhibits both biofilms, while the remaining derivatives were more effective against the biofilm of MRSA 831. The existence of persister cells, which are dormant and extremely resistant to antibiotics, intensifies the challenges posed by biofilm-associated infections. Since these derivatives suppressed the growth of the persister cells, the inhibition of the persister cells associated with the biofilms was also investigated. As shown in **Figure 5**, derivatives NPS (1–5) could inhibit 50% of persister cells attached to the biofilm in a dose and time-dependent manner. The results show that 2,2’-Bipy derivatives effectively inhibit persister cells, disrupt mature biofilms, and prevent biofilm development in Gram-positive and Gram-negative bacteria. These compounds are intriguing options for treating infections linked to biofilms because of their broad-spectrum activity and dose-dependent efficacy. Further investigation into their molecular mechanisms, including interactions with biofilm-specific targets, could enhance understanding and support their clinical translation.

#### 3.3.7 Antibacterial mechanism

**Figure 6:**
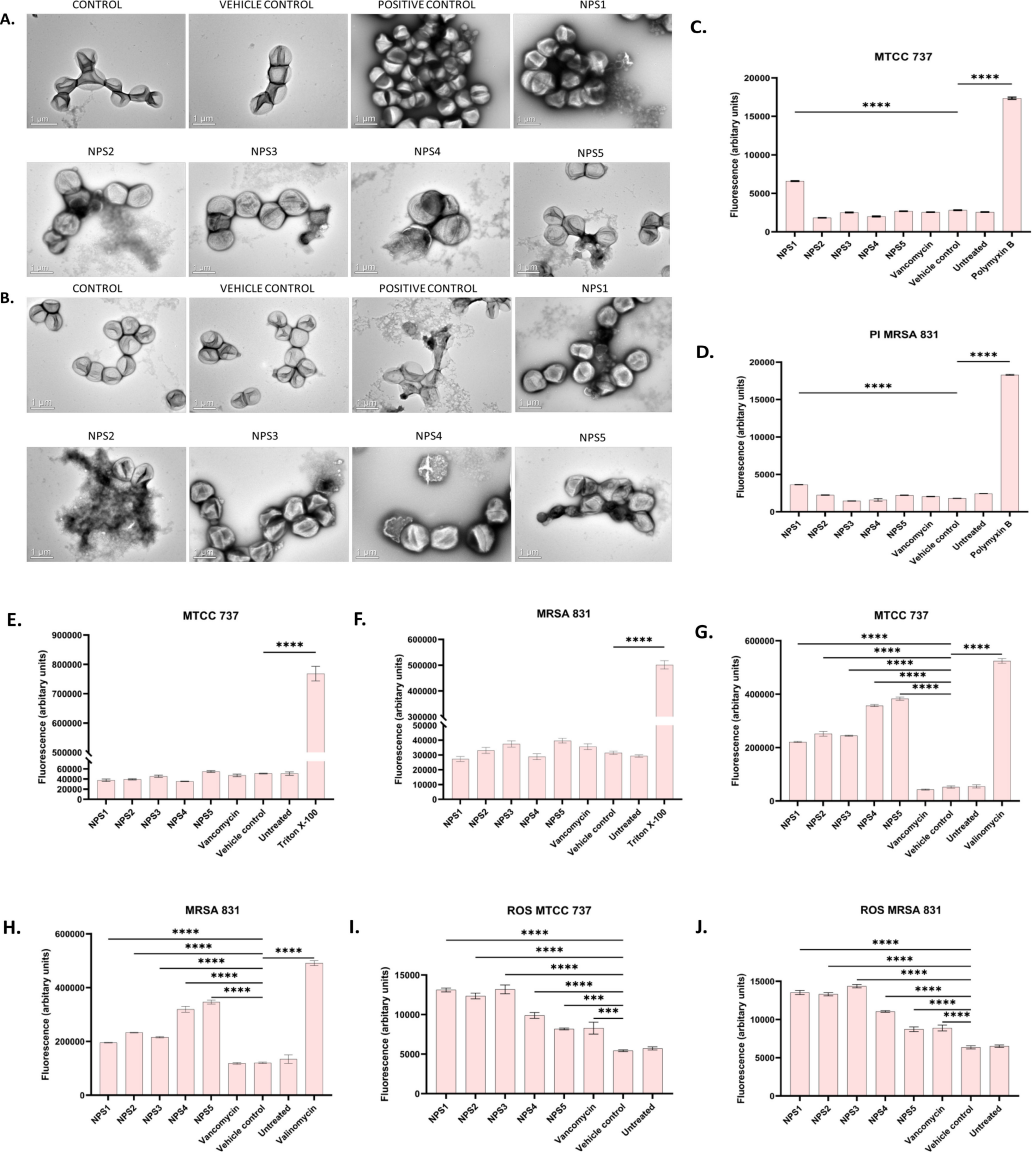
**(A, B)** Representative TEM micrographs of MTCC 737 and MRSA 831; **(C, D)** Bacterial membrane permeability of MTCC 737 and MRSA 831 using PI dye; **(E, F)** Assessment of membrane permeability with NPN dye; **(G, H)** 2,2’-Bipy derivatives depolarize the bacterial membrane of MTCC 737 and MRSA 831; **(I, J)** Total ROS accumulation in MTCC 737 and MRSA 831. Results are the cumulative data of three independent experiments (n=3) that are plotted in the graph, expressed as Mean ± SD. The p-values of 0.001 (***) and 0.0001 (****) considered highly significant.

Transmission electron microscopy (TEM) was performed to visualize the changes in cell morphology. TEM micrographs of untreated and 0.5% DMSO (vehicle control)-treated bacterial cells of *S. aureus* and MRSA 831 demonstrated typical morphology. These cells were normally spherical, with intact and smooth cell surfaces, a homogeneous shape, an intact inner membrane with its waved pattern, a uniformly thin periplasmic space, and a complete outer surface. Cells treated with 100 µg/ml vancomycin exhibited distorted morphology, wrinkled surfaces with some appendages, blebbing, buds, and a slight increase in size and irregular shape. Cells treated with derivatives at 2-fold MIC (100 µg/ml) showed symptoms of cell damage, such as missing or cracked cell walls leading to a distorted shape. Cell death commenced as the cell membrane gradually broke down, and the cytoplasm began to leak out. Certain cells appeared to be destroyed, and the majority showed clear signs of damaged and distorted membranes, with a rougher surface than the control group. Significant morphological alterations were observed in the cells treated with NPS 4 (**Figures 6A, B**). Following the detection of changes in the cell membranes of MTCC 737 and MRSA 831, the dye propidium iodide (PI) dye was utilized to investigate the antibacterial mechanism of action in order to check the membrane integrity. Disruption of bacterial membranes is one of the key steps in inhibiting bacterial growth. PI is a cell impermeable dye that penetrates the cell when the membrane is damaged. Polymyxin B, a membrane destabilizer used as a positive control, showed higher fluorescence (**Figures 6C, D**) at an excitation of 490 nm and 635 nm emission than the 2,2’-Bipy derivatives, except for NPS1, which exhibited fluorescence indicating membrane instability of the MTCC 737 and MRSA 831 bacterial cell membrane. The membrane permeability of the 2,2’-Bipy derivatives to the inner membrane of MTCC 737 and MRSA 831 was examined using NPN, a hydrophobic dye. When the derivatives were administered to the cells, the fluorescence decreased in comparison to the positive control, Triton X-100 (**Figures 6E, F**), suggesting that the derivatives don’t permeabilize the cell membrane, possibly due to limited exposure to the derivatives. Bacteria are inherently negatively charged and allow an exchange of positive ions. The nature of the charge on the bacterial membrane changes due to depolarization. The cationic dye binds to the negatively charged membrane of the polarized cells, and depolarization prevents the binding and release of dye into the medium. As a result, the control cells and the vehicle control-treated cells (not treated with drugs) produce a weak signal. In contrast, depolarized cells produced high fluorescence (**Figures 6G, H**). When derivatives were added to the cells at a concentration of 100 µg/ml (about twice the MIC), the cells exhibited intense fluorescence emission, indicating depolarization of the cell membrane. DCFH-DA is a cell-permeable dye that binds to reactive oxygen species (ROS) and produces fluorescence when read in a fluorescence reader. DCFH-DA fluorescence increased when the bacterial cells of MTCC 737 and MRSA 831 were treated with the 2,2’-Bipy derivatives compared to the vehicle control and untreated cells, indicating increased intracellular ROS (**Figures 6I, J**). Consequently, it turns out that an increase in intracellular ROS causes a shift in membrane polarity, which triggers membrane disruption by the NPS derivatives (1-5).

#### 3.3.8 Resistance development in strains MTCC 737 and MRSA 831

**Figure 7:**
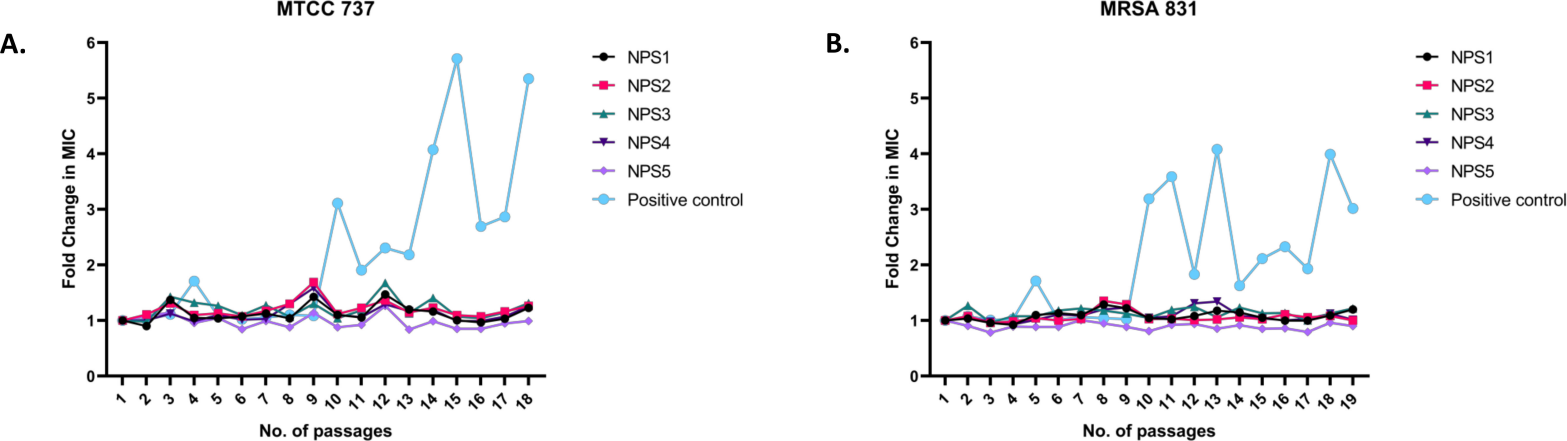
Resistance development in **(A)** MTCC 737 and **(B)** MRSA 831 after treatment with 0.5 × MIC of 2,2’-Bipy derivatives and vancomycin.

The results of resistance development using the sequential passage technique are shown in **Figure 7**. MTCC 737 and MRSA 831 were cultured for 18-19 days in the presence of 2,2’-Bipy derivatives and vancomycin. Fold change in MIC was calculated to assess resistance development. For 2,2’-Bipy derivatives, the fold change in MIC remained nearly 1 after 19 days, indicating no significant resistance development. In contrast, vancomycin treatment did not show any resistance until the 9^th^ day in both strains; after that, the fold change became neither linear nor consistent. Vancomycin resistance in various strains is well-documented in the literature. Our findings also indicate that the cells did not acquire resistance to the compounds NPS (1-5) for up to 19 days, highlighting the potential of 2,2’-Bipy derivatives in combating bacterial infections without promoting resistance. The bacteria showed no evidence of resistance, highlighting the potential of 2,2’-Bipy derivatives for managing bacterial infections, particularly in an era where antimicrobial resistance is a growing global threat.

### 3.4 *In vivo* studies

#### 3.4.1 Acute oral toxicity

Acute oral toxicity of 2,2’-Bipy derivatives was evaluated after administration of a single dose of 300 mg/kg in mice, examining behavioral, physical, hematological, biochemical, and histopathological changes. Behavioral parameters, including changes in skin, fur, and eyes, urine color, secretions from the mucous membrane, salivation, fecal consistency, autonomic behavior such as somatomotor function, and sleep, were normal. Symptoms like itching, diarrhea, tremors, and convulsions were absent. No significant differences in body weight were observed after dosing with 2,2’-Bipy derivatives. After 14 days, the mice were sacrificed, their organs collected and body weights recorded. The organ weights of derivative-treated mice were comparable to those of control and vehicle (0.5% CMC)-treated mice. All hematological parameters remained within the normal range in the test mice, with p>0.05 compared to the control group. The biochemical profiles, LFT, RFT, sugar, calcium, and lipid levels were also within the normal range compared to the control. Histopathological examination revealed no notable alterations under the light microscope (**Supplementary Figure S4**). There was no evidence of apoptosis in the liver images. The glomeruli and tubules of the kidney were in normal histological integrity. The typical histological architecture of the spleen, brain, heart, and lungs was observed, indicating no adverse effects of the 2,2’-Bipy derivatives. The lack of toxicity of these derivatives at therapeutic doses suggests a favorable safety margin, which is essential for clinical translation.

#### 3.4.2 Thigh infection model

**Figure 8:**
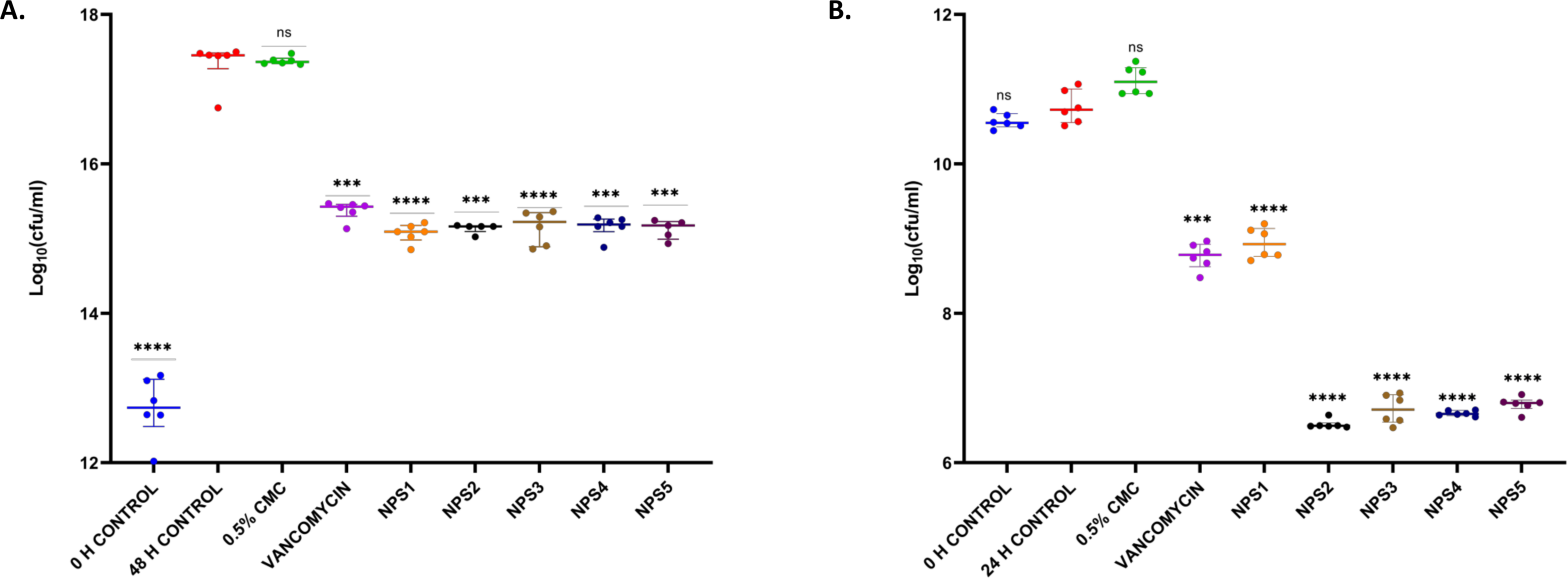
*In vivo* efficacy of 2,2’-Bipy derivatives in mice (n=6) showing bacterial burden in neutropenic thigh infection model with **(A)** MTCC 737 and **(B)** MRSA 831. The p-values of 0.001 (***) and 0.0001 (****) considered highly significant. “ns”: not significant.

In a study involving neutropenic mice infected with 10^6^ CFU/ml MTCC 737 and 10^8^ CFU/ml MRSA 831, the bacterial load in the thigh was measured following oral treatment with the derivatives NPS1, NPS2, NPS3, NPS4, NPS5, and vancomycin at 30 mg/kg. The vehicle-treated group showed a negligible reduction of -0.03 log10 in mice infected with MTCC 737, indicating minimal effect of the vehicle. Vancomycin and 2,2’-Bipy derivatives showed a reduction of approximately 1.9 log10 and 2.1-2.2 log10, respectively, in the bacterial load in tissue for the inoculum of 10^6^ CFU/ml of MTCC 737 compared to the untreated group, indicating significant efficacy after 48 h treatment (**Figure 8A**). For mice infected with MRSA 831, vancomycin and NPS1 showed a reduction of approximately1.9 log10 and ~ 1.8 log10, respectively, while NPS2-5 exhibited a greater reduction of around 4.0 log10 compared to the untreated control after 2 h and 24 h (**Figure 8B**). These results suggest that the 2,2’-Bipy derivatives, particularly NPS2-5, effectively reduce the bacterial load and show greater efficacy against MRSA 831 addressing a key limitation of vancomycin in treating multidrug-resistant infections. Additionally, the favorable safety profile of NPS2-5 and its potential to overcome resistance mechanisms highlight its promise as a next-generation antibacterial agent. These findings pave the way for further preclinical development and clinical translation of 2,2’-Bipy derivatives. The exceptional efficacy of these derivatives was evidenced by their significant reduction of bacterial load in the thigh muscles of infected mice compared to vancomycin. Specifically, they achieved greater bacterial clearance for both MTCC 737 and the clinical isolate MRSA 831, highlighting their potential as effective alternatives to traditional treatments.

## 4 Discussion

*S. aureus* is a severe and pervasive infection affecting both hospital and community settings (van Dalen et al., 2020). The rise of antibiotic-resistant strains has significantly worsened the situation, leading to the development of highly resistant bacterial variants (Founou et al., 2017). Bacteria have evolved to exist in two distinct states: the biofilm state, which is attached to a surface, and the planktonic state, where they exist as free-floating entities (Bjarnsholt et al., 2013). The U.S. National Institutes of Health (NIH) found that biofilms contribute to 65 and 80% of microbial and chronic infections, respectively, and a significant portion of infections associated with implantable devices (Mah, 2012). Biofilm’s resistance to medical treatment is largely due to its intrinsic tolerance to conventional antibiotics and host immune response (Jamal et al., 2018). This resistance is attributed to the matrix of extracellular polymeric substances (EPS) and the presence of resistant, dormant, and persistent bacteria within the biofilm matrix. The EPS matrix forms a robust, amphiphilic protective barrier, featuring a hydrophobic outer layer and a hydrophilic inner environment. This unique structure renders the biofilm highly impermeable to a wide range of chemicals, including antibiotics (Taglialegna et al., 2016; Epstein et al., 2011; Karygianni et al., 2020). Nowadays, Glycopeptide antibiotics such as vancomycin, are currently the mainstay of treatment; however, the MIC of vancomycin against MRSA strains has been increasing annually (Thakare et al., 2017). This trend is contributing to the emergence of vancomycin-resistant strains, which are leading to failure rates of vancomycin treatment for MRSA infections (Shariati et al., 2020b). When vancomycin seems to be ineffective, daptomycin is often used in combination with other drugs (including gentamicin, rifampicin, or linezolid) to treat MRSA (Hong et al., 2022). However, the emergence of resistance to both daptomycin, and vancomycin, as well as linezolid (Shariati et al., 2020a), underscore the urgent need for novel antibiotics. In response to these challenges, we evaluated the potential of 2,2’-Bipy derivatives as novel antibacterial agents. Our study assessed their efficacy both *in vitro* and *in vivo*, focusing on their ability to inhibit and eliminate biofilms.

*In silico* analysis was performed to corroborate the physiochemical and ADMET properties of 2,2’-Bipy derivatives. All six derivatives adhered to Lipinski’s rule of 5 and demonstrated an intestinal absorption rate greater than 95%, indicating that they are suitable for oral administration with minimal toxicity. Although predictions are not always experimentally validated, the calculated masses of these derivatives closely matched the experimental values obtained via mass spectrometry. Early detection of potential toxicity during the drug discovery process is critical for cost and time efficiency. Typically, an initial safety assessment of a potential antimicrobial drug involves cell culture-based *in vitro* tests, such as MTT assay or hemolysis test. To ensure the safety of these derivatives, *in vitro* experiments, including hemolysis and a cytotoxicity (MTT) assay on the 3T3L-1 cells were conducted. The 2,2’-Bipy derivatives NPS (1-6) were non-hemolytic and safe for RBCs and 3T3L-1 cells. Although vancomycin is non-hemolytic at concentrations up to 32 µg/ml, it becomes cytotoxic to normal cells at higher concentrations, with its IC_50_ value decreasing over time (Chen et al., 2011; Papalia et al., 2021). In contrast, NPS (1-6) derivatives demonstrated no toxicity in acute oral toxicity studies in mice. Conversely, vancomycin, linezolid, and daptomycin have been associated with nephrotoxicity, thrombocytopenia, and myopathy, respectively (Maddiboyina et al., 2023). The absence of toxicity at therapeutic doses for NPS (1-6) derivatives suggests a favorable safety margin, which is critical for clinical translation. The ruthenium complex containing bipyridine (Ru-3) also showed negligible cytotoxicity to mammalian red blood cells in an *in vitro* hemolysis test (Jiang et al., 2022). Furthermore, these derivatives were effective against infectious bacterial strains. They rapidly killed Gram-positive bacteria, *S. aureus*, and MRSA The MICs were calculated for *S. aureus* MTCC 737 and MRSA 831. Time-kill kinetics assays were performed for both strains and revealed a consistent decrease in absorbance compared to controls. Similar findings were reported for the ruthenium complex containing bypiridine (Ru-3), which effectively inhibited *S. aureu*s (Jiang et al., 2022). Additionally, bipyridine antibiotic compounds produced by *Saccharothrix xinjiangensis*, showed activity against several pathogenic Gram-positive bacteria, including *S. aureus* and MRSA and it was noted that the chemical structure of these compounds influenced their antimicrobial activity, with enhanced efficacy observed when the oxime group was attached to the bipyridine ring and reduced activity when sugars were present (Lahoum et al., 2019). The derivatives also proved effective against the persister cells phenotypic variants of latent bacterial cells that are resistant to conventional antibiotics and inhibited the cells are referred to as persister cells (Lewis, 2010). This study focuses on strategies for treating biofilm-associated infections, characterized by enhanced resistance to antibiotics, and host immune defenses. The study evaluates the ability of 2,2’-Bipy derivatives to inhibit biofilm formation and disrupt pre-formed biofilms, emphasizing their potential as anti-biofilm agents. Biofilms are challenging to treat due to factors such as limited antibiotic penetration through the EPS matrix, altered microenvironments within the biofilm, and the presence of persister cells. The 2,2’-Bipy derivatives demonstrated a significant ability to inhibit biofilm formation and eradicate pre-formed biofilms in a dose-dependent manner, particularly against biofilm-associated persister cells. The study also compared the efficacy of 2,2’-Bipy derivatives to vancomycin, a gold-standard antibiotic for combating MRSA infections, revealing their potential as superior alternatives in addressing biofilm-related challenges. Treatment with derivative NPS (1-5) led to dose-dependent inhibition and eradication of biofilms formed by MTCC 737 and MRSA 831. This was confirmed by visual CLSM imaging, which showed that the derivatives disrupted and damaged the biofilms. Similarly, bipyridines and collismycin C (2) inhibited MRSA biofilm formation. This inhibitory effect is probably related to iron acquisition and the presence and positioning of the hydroxyl group in 2,2’-Bipy (Lee et al., 2017).

TEM was used to observe membrane disruption and cytoplasmic leakage in *S. aureus* MTCC 737 and MRSA 831 upon exposure to the derivatives. We also conducted permeabilization, NPN uptake, depolarization, and ROS experiments to assess cell morphology and membrane integrity. Derivatives NPS (1-5) were found to increase ROS production, which led to membrane potential depolarization and membrane damage, ultimately resulting in bacterial cell death. Similarly, the biological activity of Ru-3 revealed its effectiveness in preventing biofilm formation and disrupting cell membranes (Jiang et al., 2022). The lipophilic Ga complex [Ga_2_L_3_(bpy)_2_], which consists of 2,2’-Bipy, also exhibited increased intracellular ROS in *S. aureus*, contributing to cell death (Wang et al., 2021). The development of drug resistance is a critical consideration in designing and evaluating new antimicrobial agents. To assess this, we performed resistance development tests on the 2,2’-Bipy derivatives with strains MTCC 737 and MRSA 831. Both strains developed resistance to vancomycin after 9 days; however, no resistance to the derivatives was observed over 20 days of passage. Similarly, *S. aureus* did not develop resistance to [Ga_2_L_3_(bpy)_2_] (Wang et al., 2021). The bacteria showed no evidence of resistance, highlighting the potential of the derivatives in managing bacterial infections, particularly in an era where antimicrobial resistance is a growing global threat. Additionally, the 2,2’ Bipy derivatives demonstrated efficacy in neutropenic thigh infection mice against MTCC 737 and MRSA 831. The remarkable efficacy of these derivatives was indicated by the reduction of bacterial load of MTCC 737 and clinical isolate MRSA 831 in the thigh muscles of infected mice compared to vancomycin. The favorable safety profile of NPS2-5, coupled with its efficacy to address resistance mechanisms, highlights its potential as a next-generation antibacterial agent. The derivatives effectively inhibited biofilm formation, eradicated pre-formed biofilms and targeted persister cells, outperforming vancomycin in certain assays. Future research should focus on elucidating the mechanisms by which these derivatives interact with biofilm-specific targets, disrupt the EPS matrix, and eliminate persister cells. These findings lay a strong foundation for further preclinical development and eventually pave the way for the clinical translation of 2,2’-Bipy derivatives.

## 5 Conclusion

Antimicrobial resistance is a severe problem exacerbated by the overuse of antibiotics, with MRSA being a significant global health concern due to its rapid spread among living organisms. Intermediate resistance levels hinder vancomycin’s efficacy in combating MRSA infections, necessitating advancements in anti-MRSA therapies. 2,2’-Bipy is one of the unique scaffolds that has been explored in various diseases, but it remains highly efficient when studied with modifications. This study evaluated 2,2’-Bipy derivatives with modified functional groups against *S. aureus* and MRSA Adding aryl or allyl residues to the parent 2,2’-Bipy skeleton was vital in enhancing the antibacterial activity against MRSA

In conclusion, 2,2’-Bipy derivatives exhibit significant antibacterial activity against the MRSA 831 and MTCC 737 clinical strains. They effectively reduce bacterial biofilms and demonstrate good *in vivo* efficiency, with no harmful effects in either *in vitro* or *in vivo* settings.

## Data Availability

The original contributions presented in the study are included in the article/**Supplementary Material**. Further inquiries can be directed to the corresponding author.
