# Supplementary material for: Comprehensive safety and toxicity analysis of 2,2’-Bipyridine derivatives in combating MRSA biofilm formation and persistence
Source: Front Cell Infect Microbiol. 2025 Jan 24;15:1493679. doi: 10.3389/fcimb.2025.1493679 (PMC11802822; doi:10.3389/fcimb.2025.1493679)
Supplement: Supplementary file 1 [file DataSheet1.docx]

Supplementary Material

2,2’-Bipyridine Derivatives in Confronting MRSA Biofilm Formation and Persistence

Priyanka^1,2^, Mohini Sharma^1^, Bhavna Vaid^1,3^, Ram Bharti^1,2^, Sachin Raut^1,2^, R. S. Jolly^4^ & Neeraj Khatri^1,2^*

^1^IMTECH Centre for Animal Resources & Experimentation (iCARE), Council of Scientific and Industrial Research-Institute of Microbial Technology, Sector 39-A, Chandigarh-160036, India

^2^Academy of Scientific and Innovative Research, Ghaziabad-201002, India

^3^PG Department of Chemistry, Sri Guru Tegh Bahadur Khalsa College, Sri Anandpur Sahib, Punjab, India

^4^Council of Scientific and Industrial Research-Institute of Microbial Technology, Sector 39-A, Chandigarh-160036, India

* Corresponding author-

Neeraj Khatri,

Email: neeraj@imtech.res.in

**Running title:** Anti-biofilm and anti-persister potential of 2,2’-Bipyridine derivatives

**Supplementary materials**


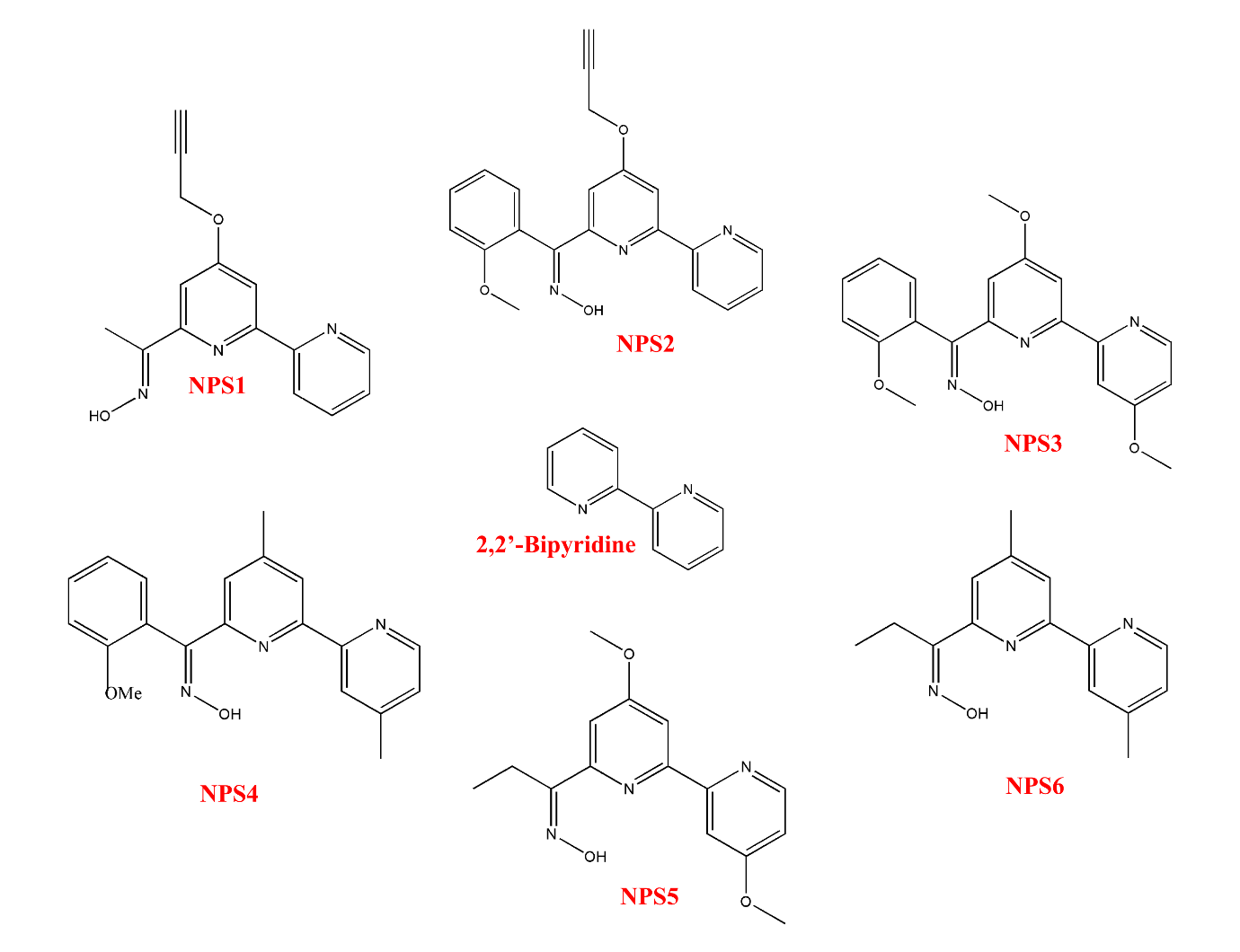


**Fig. S1.** Chemical structures of derivatives with 2,2’-bipyridine scaffold.


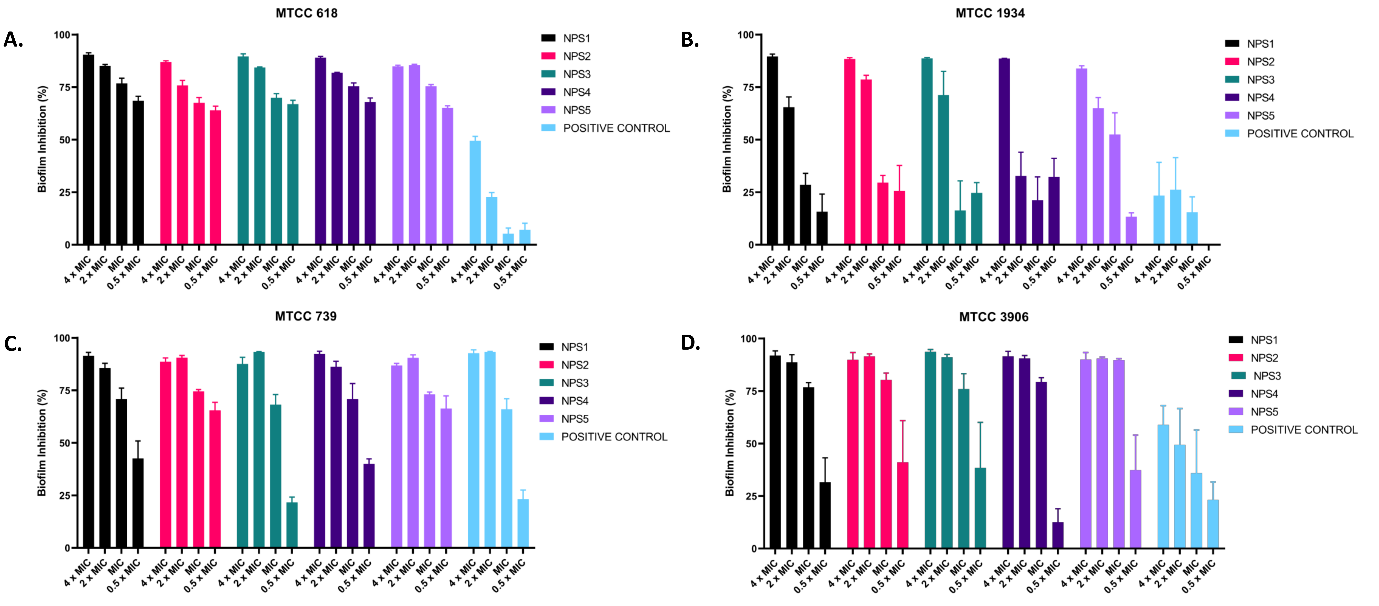


**Fig. S2.** (A-D) Biofilm inhibition against *K. pneumoniae* (MTCC 618), *P. aeruginosa* (MTCC 1934), *E. coli* (MTCC 739), and *V. cholerae* (MTCC 3906) at four different concentrations 0.5- 4 X MIC of 2,2’-bipyridine derivatives and Vancomycin


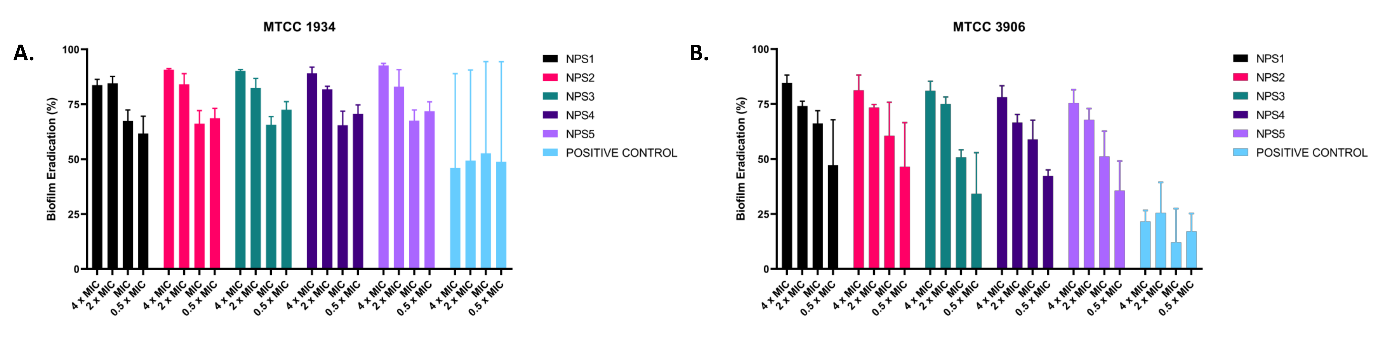


**Fig. S3.** (A-B) Biofilm eradication against *P. aeruginosa* (MTCC 1934) and *V. cholerae* (MTCC 3906) at four different concentrations 0.5- 4 X MIC of 2,2’-bipyridine derivatives and Vancomycin.


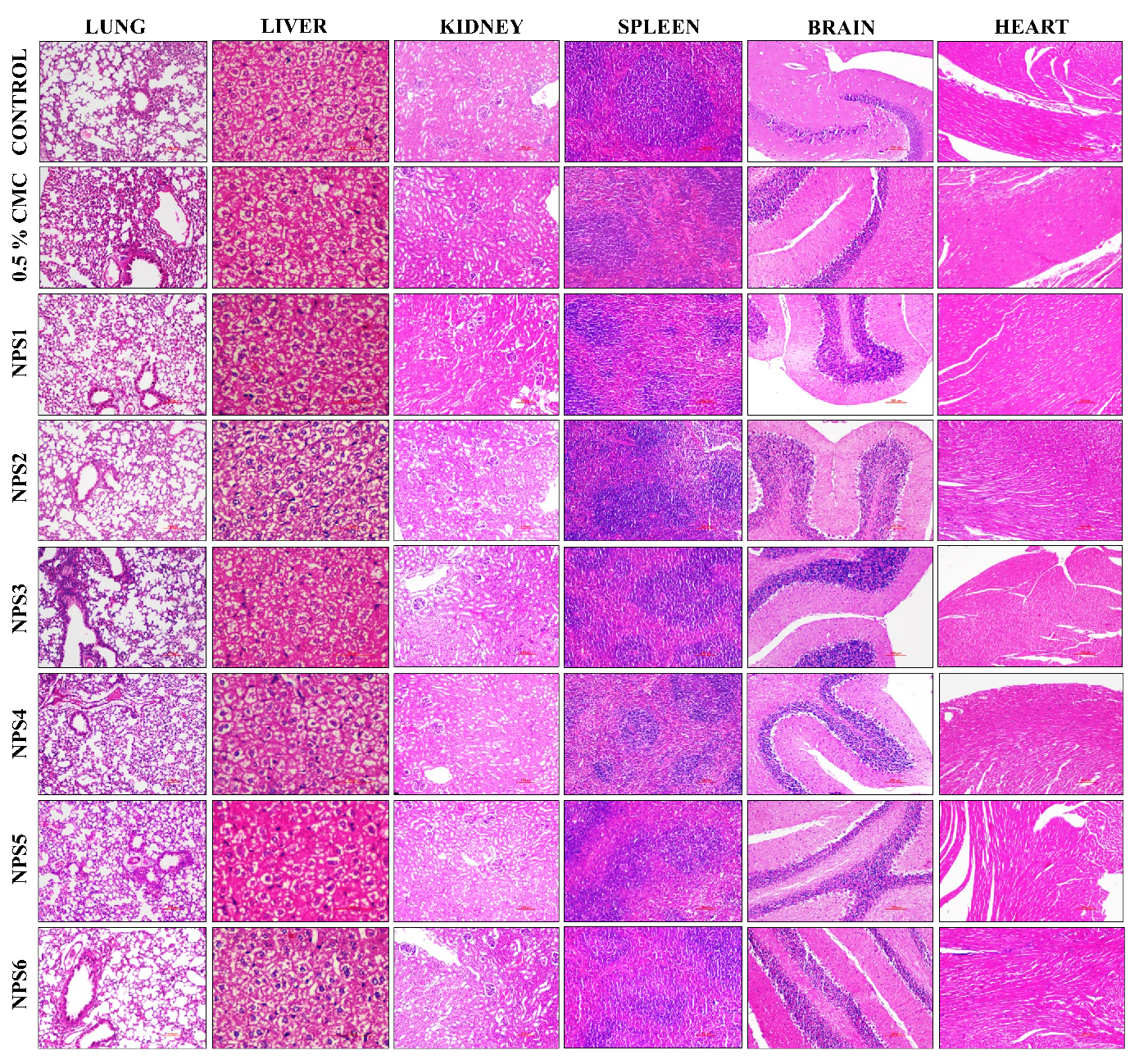


**Fig. S4.** Representative histograph of various organs of C57BL6 female mice in an acute oral toxicity study (n=3).
